# Genomic influences on self-reported childhood maltreatment

**DOI:** 10.1038/s41398-020-0706-0

**Published:** 2020-01-27

**Authors:** Shareefa Dalvie, Adam X. Maihofer, Jonathan R. I. Coleman, Bekh Bradley, Gerome Breen, Leslie A. Brick, Chia-Yen Chen, Karmel W. Choi, Laramie E. Duncan, Guia Guffanti, Magali Haas, Supriya Harnal, Israel Liberzon, Nicole R. Nugent, Allison C. Provost, Kerry J. Ressler, Katy Torres, Ananda B. Amstadter, S. Bryn Austin, Dewleen G. Baker, Elizabeth A. Bolger, Richard A. Bryant, Joseph R. Calabrese, Douglas L. Delahanty, Lindsay A. Farrer, Norah C. Feeny, Janine D. Flory, David Forbes, Sandro Galea, Aarti Gautam, Joel Gelernter, Rasha Hammamieh, Marti Jett, Angela G. Junglen, Milissa L. Kaufman, Ronald C. Kessler, Alaptagin Khan, Henry R. Kranzler, Lauren A. M. Lebois, Charles Marmar, Matig R. Mavissakalian, Alexander McFarlane, Meaghan O’ Donnell, Holly K. Orcutt, Robert H. Pietrzak, Victoria B. Risbrough, Andrea L. Roberts, Alex O. Rothbaum, Peter Roy-Byrne, Ken Ruggiero, Antonia V. Seligowski, Christina M. Sheerin, Derrick Silove, Jordan W. Smoller, Murray B. Stein, Martin H. Teicher, Robert J. Ursano, Miranda Van Hooff, Sherry Winternitz, Jonathan D. Wolff, Rachel Yehuda, Hongyu Zhao, Lori A. Zoellner, Dan J. Stein, Karestan C. Koenen, Caroline M. Nievergelt

**Affiliations:** 1 SA MRC Unit on Risk & Resilience in Mental Disorders, Department of Psychiatry and Mental Health, University of Cape Town, Cape Town, South Africa; 2 Department of Psychiatry, University of California San Diego, La Jolla, CA USA; 3 Veterans Affairs San Diego Healthcare System, Center of Excellence for Stress and Mental Health, San Diego, CA USA; 4 Veterans Affairs San Diego Healthcare System, Research Service, San Diego, CA USA; 5 King’s College London, Social, Genetic and Developmental Psychiatry Centre, Institute of Psychiatry, Psychology and Neuroscience, London, UK; 6 King’s College London, NIHR BRC at the Maudsley, London, UK; 7 Atlanta VA Health Care System, Mental Health Service Line, Decatur, GA USA; 8 Department of Psychiatry and Behavioral Sciences, Emory University, Atlanta, GA USA; 9 Alpert Medical School of Brown University, Providence, RI USA; 10 Massachusetts General Hospital, Analytic and Translational Genetics Unit, Boston, MA USA; 11 Massachusetts General Hospital, Psychiatric and Neurodevelopmental Genetics Unit (PNGU), Boston, MA USA; 12 Broad Institute of MIT and Harvard, Stanley Center for Psychiatric Research, Cambridge, MA USA; 13 Harvard T.H. Chan School of Public Health, Department of Epidemiology, Boston, MA USA; 14 Massachusetts General Hospital, Department of Psychiatry, Boston, MA USA; 15 Department of Psychiatry and Behavioral Sciences, Stanford University, Stanford, CA USA; 16 Harvard Medical School, Department of Psychiatry, Boston, MA USA; 17 McLean Hospital, Belmont, MA USA; 18 Cohen Veterans Bioscience, Cambridge, MA USA; 19 Department of Psychiatry, University of Michigan Medical School, Ann Arbor, MI USA; 20 Bradley/Hasbro Children’s Research Center of Rhode Island Hospital, Providence, RI USA; 21 Virginia Institute for Psychiatric and Behavioral Genetics, Department of Psychiatry, Richmond, VA USA; 22 Harvard Medical School, Department of Psychiatry, Boston, MA USA; 23 Harvard School of Public Health, Department of Social and Behavioral Sciences, Boston, MA USA; 24 Boston Children’s Hospital, Division of Adolescent and Young Adult Medicine, Boston, MA USA; 25 Brigham and Women’s Hospital, Channing Division of Network Medicine, Boston, MA USA; 26 Veterans Affairs San Diego Healthcare System, Psychiatry Service, San Diego, CA USA; 27 Department of Psychology, University of New South Wales, Sydney, NSW Australia; 28 Department of Psychiatry, University Hospitals, Cleveland, OH USA; 29 Department of Psychological Sciences, Kent State University, Kent, OH USA; 30 Research and Sponsored Programs, Kent State University, Kent, OH USA; 31 Department of Medicine, Boston University School of Medicine, Boston, MA USA; 32 Department of Psychological Sciences, Case Western Reserve University, Cleveland, OH USA; 33 Department of Psychiatry, Icahn School of Medicine at Mount Sinai, New York, NY USA; 34 Department of Psychiatry, University of Melbourne, Melbourne, VIC Australia; 35 Department of Psychological and Brain Sciences, Boston University, Boston, MA USA; 36 US Army Medical Research and Materiel Command, Fort Detrick, MD USA; 37 US Department of Veterans Affairs, Department of Psychiatry, West Haven, CT USA; 38 VA Connecticut Healthcare Center, West Haven, CT USA; 39 Department of Genetics and Neuroscience, Yale University School of Medicine, New Haven, CT USA; 40 US Army Medical Research and Materiel Command, USACEHR, Fort Detrick, MD USA; 41 Harvard Medical School, Department of Health Care Policy, Boston, MA USA; 42 Department of Psychiatry, University of Pennsylvania Perelman School of Medicine, Philadelphia, PA USA; 43 Mental Illness Research, Education and Clinical Center, Crescenz VAMC, Philadelphia, PA USA; 44 Department of Psychiatry, New York University School of Medicine, New York, NY USA; 45 University of Adelaide, Centre for Traumatic Stress Studies, Adelaide, SA Australia; 46 Department of Psychology, Northern Illinois University, DeKalb, IL USA; 47 U.S. Department of Veterans Affairs National Center for Posttraumatic Stress Disorder, West Haven, CT USA; 48 Department of Psychiatry, Yale University School of Medicine, New Haven, CT USA; 49 Department of Environmental Health, Harvard T.H. Chan School of Public Health, Boston, MA USA; 50 Department of Psychiatry, University of Washington, Seattle, WA USA; 51 Department of Nursing and Department of Psychiatry, Medical University of South Carolina, Charleston, SC USA; 52 Department of Psychiatry, University of New South Wales, Sydney, NSW Australia; 53 Veterans Affairs San Diego Healthcare System, Million Veteran Program, San Diego, CA USA; 54 Department of Psychiatry, Uniformed Services University, Bethesda, MD USA; 55 Department of Mental Health, James J. Peters VA Medical Center, Bronx, NY USA; 56 Department of Biostatistics, Yale University, New Haven, CT USA; 57 Department of Psychiatry and Behavioral Sciences, University of Washington, Seattle, WA USA; 58 Harvard School of Public Health, Department of Epidemiology, Boston, MA USA

**Keywords:** Medical genetics, Psychiatric disorders

## Abstract

Childhood maltreatment is highly prevalent and serves as a risk factor for mental and physical disorders. Self-reported childhood maltreatment appears heritable, but the specific genetic influences on this phenotype are largely unknown. The aims of this study were to (1) identify genetic variation associated with self-reported childhood maltreatment, (2) estimate SNP-based heritability (*h*^2^_snp_), (3) assess predictive value of polygenic risk scores (PRS) for childhood maltreatment, and (4) quantify genetic overlap of childhood maltreatment with mental and physical health-related phenotypes, and condition the top hits from our analyses when such overlap is present. Genome-wide association analysis for childhood maltreatment was undertaken, using a discovery sample from the UK Biobank (UKBB) (*n* = 124,000) and a replication sample from the Psychiatric Genomics Consortium-posttraumatic stress disorder group (PGC-PTSD) (*n* = 26,290). *h*^2^_snp_ for childhood maltreatment and genetic correlations with mental/physical health traits were calculated using linkage disequilibrium score regression. PRS was calculated using PRSice and mtCOJO was used to perform conditional analysis. Two genome-wide significant loci associated with childhood maltreatment (rs142346759, *p* = 4.35 × 10^−8^, *FOXP1*; rs10262462, *p* = 3.24 × 10^−8^, *FOXP2*) were identified in the discovery dataset but were not replicated in PGC-PTSD. *h*^2^_snp_ for childhood maltreatment was ~6% and the PRS derived from the UKBB was significantly predictive of childhood maltreatment in PGC-PTSD (*r*^2^ = 0.0025; *p* = 1.8 × 10^−15^). The most significant genetic correlation of childhood maltreatment was with depressive symptoms (*r*_g_ = 0.70, *p* = 4.65 × 10^−40^), although we show evidence that our top hits may be specific to childhood maltreatment. This is the first large-scale genetic study to identify specific variants associated with self-reported childhood maltreatment. Speculatively, *FOXP* genes might influence externalizing traits and so be relevant to childhood maltreatment. Alternatively, these variants may be associated with a greater likelihood of reporting maltreatment. A clearer understanding of the genetic relationships of childhood maltreatment, including particular abuse subtypes, with a range of phenotypes, may ultimately be useful in in developing targeted treatment and prevention strategies.

## Introduction

The lifetime prevalence of childhood physical, sexual, and emotional maltreatment ranges from 8% to 36%^1^. In addition to being highly prevalent, such childhood abuse is associated with the development of mental disorders, including depression^2,3^, and physical ill health, including non-communicable diseases^4,5^. Although these associations are now well established, estimates of effect size vary considerably across epidemiological studies, likely reflecting methodological challenges, including uncertainty about how best to assess childhood maltreatment^6^.

A twin-based study found that retrospective reports of childhood maltreatment has a heritability of 6%^7^. Although the idea that childhood maltreatment is heritable may seem counter-intuitive, work on behavior genetics has long documented the heritability of many exposures perceived as environmental. Such heritability is referred to as gene–environment correlation (rGE), and three potential rGE mechanisms to explain the heritability of childhood maltreatment may be posited. First, a “passive” rGE: parental genes affecting parental behavior may influence the childhood environment (e.g. aggressive parents may be more likely to physically punish their children^8^). Second, an “active” rGE: individuals with genetic variants associated with certain behavioral phenotypes may be more at risk of selecting or creating adverse situations (e.g. risk-taking is heritable and children who are high in risk-taking may be exposed to more trauma)^9,10^. Third, an “evocative” rGE: genetic variation may influence child behavior, which in turn is associated with responses to the child (e.g. genetic factors may influence infant “difficultness”, which in turn is associated with maternal hostile-reactive behavior that is correlated with child abuse^11,12^). The latter two rGEs are sometimes collectively referred to as non-passive correlations^7^.

While a number of key risk factors for childhood maltreatment, including child behavioral characteristics and parental mental health, have been investigated^6^, studies have seldom focused on associated genetic variation. The few genetic association studies of childhood maltreatment have only considered variants in candidate genes^13^ and have had insufficient power to detect the small polygenic effect sizes typically associated with behavioral phenotypes^14^. Also, there are no studies of the genetic overlap of childhood maltreatment with mental and physical health-related traits, using genome-wide single nucleotide polymorphism (SNP) data. Knowledge of specific genetic variation for childhood maltreatment, the heritability of this phenotype, the polygenic risk, and the genetic overlap with other traits may be useful in informing our understanding of the risk factors, the etiology, and the outcomes of childhood maltreatment. This, in turn, may have implications for the design of prevention and treatment programs for adverse health outcomes. For example, environmental exposures that play a causal role in impacting health outcomes are likely to mediate any observed associations between genetic variants and that health outcome (e.g. early loss of a parent may lead to depression, with such loss then mediating the association between heritability of early parental loss and depression). Thus, preventative strategies would focus on decreasing the risk conferred by the environmental exposure without needing to specifically consider the genetic influences on the health outcome^9^ (e.g. development of programs for children who have experienced early loss).

The PGC-PTSD has collaborated to obtain access to well-powered genetic studies of trauma and PTSD that have allowed a number of key genetic questions in this field to be investigated^15–17^, providing a unique opportunity to address knowledge gaps in the area of childhood maltreatment. This study aims to: (1) identify genetic variants associated with childhood maltreatment using a genome-wide association study (GWAS) design, (2) quantify the heritability of childhood maltreatment using SNP-based methods, (3) assess the predictive value of polygenic risk scores (PRS) for childhood maltreatment, and (4) assess the degree of genetic overlap of childhood maltreatment with mental and physical health-related phenotypes, and condition the top genome-wide hits from our analyses when such overlap is present.

## Materials and methods

### Participating studies

Nineteen studies, comprising subjects of European ancestry only, were used in this analysis. The discovery dataset consisted of 124,711 individuals with available childhood maltreatment data from the UK Biobank (UKBB)^18^, and the replication sample comprised 26,290 individuals—a subset of the PGC-PTSD Freeze 1.5 dataset (PGC1.5)^17^. The details of these studies, including the demographics and instruments used to assess maltreatment can be found in Supplementary Table 1. We have complied with relevant ethical regulations for work with human subjects. All subjects provided written informed consent and studies were approved by the relevant institutional review boards and the UCSD IRB (protocol #16097×).

### Phenotype harmonization

For the childhood maltreatment phenotype, Childhood Trauma Questionnaire (CTQ) scores on physical, sexual, and emotional abuse^19^ were obtained from the participating studies. From this, an overall childhood maltreatment count score of 0–3 was constructed, based on a count of the three abuse categories listed above. An individual was considered to have endorsed a childhood abuse category if they scored in the moderate to extreme range for that particular category, per established cut-offs^20^ (Supplementary Table 2). If CTQ data were not available, the event assessment during childhood (occurring before 18 years of age) that was most validated for that particular study was obtained, providing a count of the total number of different categories of reported childhood events (e.g. physical, sexual, or severe emotional abuse) along with the range of possible scores for the measure. The reported maltreatment exposure from the UKBB dataset comprised a score of three items where participants were asked whether they were (i) “physically abused by family as a child”, (ii) “sexually molested as a child”, and whether they (iii) “felt hated by family member as a child”. The childhood maltreatment count score, whether it was generated from the CTQ or another instrument, was used as the main outcome measure in the association analysis. The range and mean of maltreatment count scores for each study can be seen in Supplementary Table 1.

### Global ancestry determination, genotyping quality control, and imputation

Study participants from the PGC-PTSD were genotyped with a number of different arrays (Supplementary Table 1). Genotype data were quality controlled and processed using the standard PGC pipeline, Ricopili-MANC (https://sites.google.com/a/broadinstitute.org/ricopili/ and https://github.com/orgs/Nealelab/teams/ricopili) as part of the PGC-PTSD Freeze 2 data analysis^17,21^. This work was carried out on the Dutch national e-infrastructure with the support of SURF Cooperative. A detailed outline of these methods can be found in ref. ^17^. Briefly, ancestry was determined with pre-QC genotypes using a SNPweights panel of 10,000 ancestry informative markers from a reference panel comprising 2911 subjects from 71 diverse populations and six continental groups (https://github.com/nievergeltlab/global_ancestry). Samples with estimated > 90% European ancestry were classified as European. Samples were excluded if they had call rates < 98%, deviated from the expected inbreeding coefficient (fhet < −0.2 or >0.2), or had a sex discrepancy between reported and genotypic sex (based on inbreeding coefficients calculated from SNPs on the X chromosome). Markers were excluded if they had call rates < 98%, >2% difference in missing genotypes between PTSD cases and controls, or were monomorphic. Markers with a Hardy–Weinberg equilibrium (HWE) *p* < 1 × 10^−6^ in controls were excluded from all subjects. Principal components (PCs) were calculated using the smartPCA algorithm in EIGENSTRAT^22^. Pre-phasing and phasing was performed using SHAPEIT2 v2.r837^23^. Imputation was performed with IMPUTE2 v2.2.2^24^ using the 1000 Genomes (1000G) phase 3 data^25^ as the reference.

Details regarding the QC, imputation, and ancestry determination of the UKBB dataset can be found in ref. ^26^. Briefly, study participants were genotyped with two custom genotyping arrays (with ∼800,000 markers). A two-stage imputation was performed using the Haplotype Reference Consortium (HRC)^27^ and the UK10K^28^ as the reference panels. Variants in the UKBB dataset were filtered to include only those with a minor allele frequency (MAF) of > 1% and an INFO score of > 0.4. Related individuals (third degree and closer) and those with a genotyping call rate < 98% were excluded. Ancestry was determined by 4-means clustering on the first two PCs provided by the UKBB^29^. Additional principal component analysis was conducted on the European-only data subset using flashpca2^30^.

### Main GWAS

GWAS analysis was conducted separately for each study. Best-guess genotypes were tested for association to self-reported childhood maltreatment using an ordinal logistic regression model with age, sex, and the first five PCs included as covariates. Variants with a MAF < 0.5% and a genotyping rate < 98% were excluded, for all studies except the UKBB. These analyses were implemented in PLINK 1.9^31^ using the plug-in *Rserve*. To ensure computational efficiency, linear regression models were run for 4 of the larger contributing studies (NSS1; NSS2; PPDS; and UKBB, *N* = 143,392 subjects)^17^. For the NSS1; NSS2; and PPDS studies, age, sex, and 5 PCs were included as covariates in the regression model. For the UKBB dataset, the regression analysis was implemented in BGenie v1.2^32^ with age, sex, 6 PCs, batch, and site included as covariates. All tests performed were two-sided.

### Meta-analysis

As both linear and ordinal logistic models were implemented in the GWASs, which resulted in different effect statistics, fixed effects meta-analysis was conducted across studies using *p*-values and direction of effect, weighted according to the effective sample size as the analysis scheme, in METAL (v. March 25 2011)^33^. Effective sample sizes (*N*_eff_) for ordinal logistic regressions were calculated as *N*_eff_ *=* harmonic mean*n levels of childhood maltreatment, and for linear regressions as *N*_eff_ *=* ((1−probability of having a zero score) × mean of nonzero data)^34^. Heterogeneity across datasets was tested using the Cochran’s Q-test for heterogeneity, also implemented in METAL. Only variants with an INFO score of >0.8 and a conservative MAF of >5% were included in the meta-analysis, except where otherwise indicated in the results. Forest plots were generated for genome-wide significant hits using the R package *meta*^35^.

### Functional mapping and annotation

Genome-wide significant hits identified from the GWAS meta-analysis were annotated using the web-based tool FUnctional Mapping and Annotation (FUMA) v1.3.4c^36^. Default settings were used and annotations were based on the human genome assembly GRCh37 (hg19). The *SNP2GENE* module was used to identify genomic risk loci and these were mapped to protein-coding genes within a 10 kb window. An *r*^2^ of ≥ 0.6 was used to identify variants in LD with lead SNPs. The 1000G European Phase 3 was used as the reference dataset. Variants were functionally annotated using ANNOVAR, combined dependent depletion (CADD), RegulomeDB (RDB), and chromatin states (only tissues/cells from brains were included). The NHGRI-EBI GWAS catalog was used to determine any previous associations with the identified risk variants. The GTEx v7 brain tissue, RNAseq data from the CommonMind Consortium and the BRAINEAC database were used to perform eQTL mapping for significant SNP–gene pairs (FDR *q* < 0.05).

A gene-based analysis was performed within FUMA using MAGMA whereby SNPs were mapped to 18,989 protein-coding genes. Genome-wide significance was set at a Bonferroni-corrected threshold *p* < 2.63 × 10^−6^. In addition, gene-based test statistics were used to determine whether specific biological pathways are associated with childhood maltreatment. This was performed for 10,678 curated gene sets and GO terms obtained from MsigDB, using MAGMA. The significance threshold was set at a Bonferroni-corrected threshold of *p* = 4.68 × 10^−6^ (0.05/10,678).

### Heritability estimation

Linkage disequilibrium score regression (LDSR) is a technique for quantifying polygenicity and confounding, such as population stratification, in GWAS summary statistics^37^. This is accomplished by evaluating the relationship between linkage disequilibrium (LD) scores (the average squared correlation of a SNP with all neighboring SNPs) and SNP test statistics. Using this approach, the LDSR intercept was used to estimate the proportion of inflation in test statistics due to polygenic signal (rather than inflation due to population stratification and cryptic relatedness), with the Eq. (1)*—*(LDSR intercept−1)/(mean observed chi-square−1)^17^. Using GWAS summary statistics, SNP-based heritability (*h*^2^_snp_) was calculated, which is one of the applications of LDSR.

### Polygenic risk scoring

Using PRSice v2.1.3.beta^38^, PRS were calculated in target samples (PGC1.5) based on SNP effect sizes from childhood maltreatment GWAS in non-overlapping discovery/training samples (UKBB). Multiple *p*-value thresholds (*P*_T_) (0.001, 0.05, 0.1, 0.2, 0.3, 0.4, 0.5, 1) were generated using the best guess genotype data of target samples. Variants with a MAF < 5% were excluded from the discovery dataset. As a default in PRSice, LD pruning was performed whereby variants were pruned if they were nearby (within 250 kb) and in LD (*r*^2^ > 0.1) with the leading variant (lowest *p*-value) in a given region. For this analysis, a rescaled childhood maltreatment phenotype was generated whereby the childhood maltreatment score for each individual was divided by the theoretical maximum score for a given study. Best-fit PRS (at *P*_T_ = 0.0354) were used to predict childhood maltreatment status as a quantitative trait, adjusting for five PCs and dummy study indicator variables. As women in PGC1.5 experienced significantly more childhood maltreatment than men, we generated PRS in women and men separately. The proportion of variance explained by PRS was estimated as the difference in Nagelkerke’s *R*^2^ between the full model (which includes PRS plus covariates) and the null model (which only has the covariates). PRS prediction plots were based on quantiles of PRS, with effect sizes calculated in reference to the lowest quantile. *p*-values for PRS were derived from a likelihood ratio test comparing the two models. The significance threshold was set at a Bonferroni-corrected threshold of *p* = 0.006 (0.05/8).

### Genetic correlation

Another application of LDSR is the measurement of genetic correlation, i.e. the degree and direction of shared genetic effects between different traits^37,39^. Cross-cohort genetic correlation (*r*_g_) was calculated using LDSR. The web-based interface for LDSR, LD Hub, was used to further calculate pairwise genetic correlations between childhood maltreatment and 247 non-UKBB traits of interest including psychiatric, anthropomorphic, smoking behavior, reproductive, aging, education, autoimmune, and cardio-metabolic categories.

### Conditional analyses of childhood maltreatment top hits

To evaluate if the effects of top variants in the UKBB GWAS and meta-analysis were specific to childhood maltreatment, we conditioned childhood maltreatment on genetically correlated traits using the multi-trait conditional and joint analysis (mtCOJO)^40^ feature in GCTA^41^. Data for major depressive disorder (MDD)^42^ (from https://www.med.unc.edu/pgc/results-and-downloads) was used to minimize sample overlap with the UK Biobank data. The effect of the correlated trait on childhood maltreatment was estimated using a generalized summary-data based Mendelian randomization analysis of significant LD independent SNPs (*r*^2^ < 0.05, based on 1000G Phase 3 CEU samples). The threshold for significance was set at *p* < 5 × 10^−6^, due to having less than the required 10 significant independent SNPs at the program default of *p* < 5 × 10^−8^, for the correlated trait.

## Results

### GWAS and meta-analysis

**Fig. 1:**
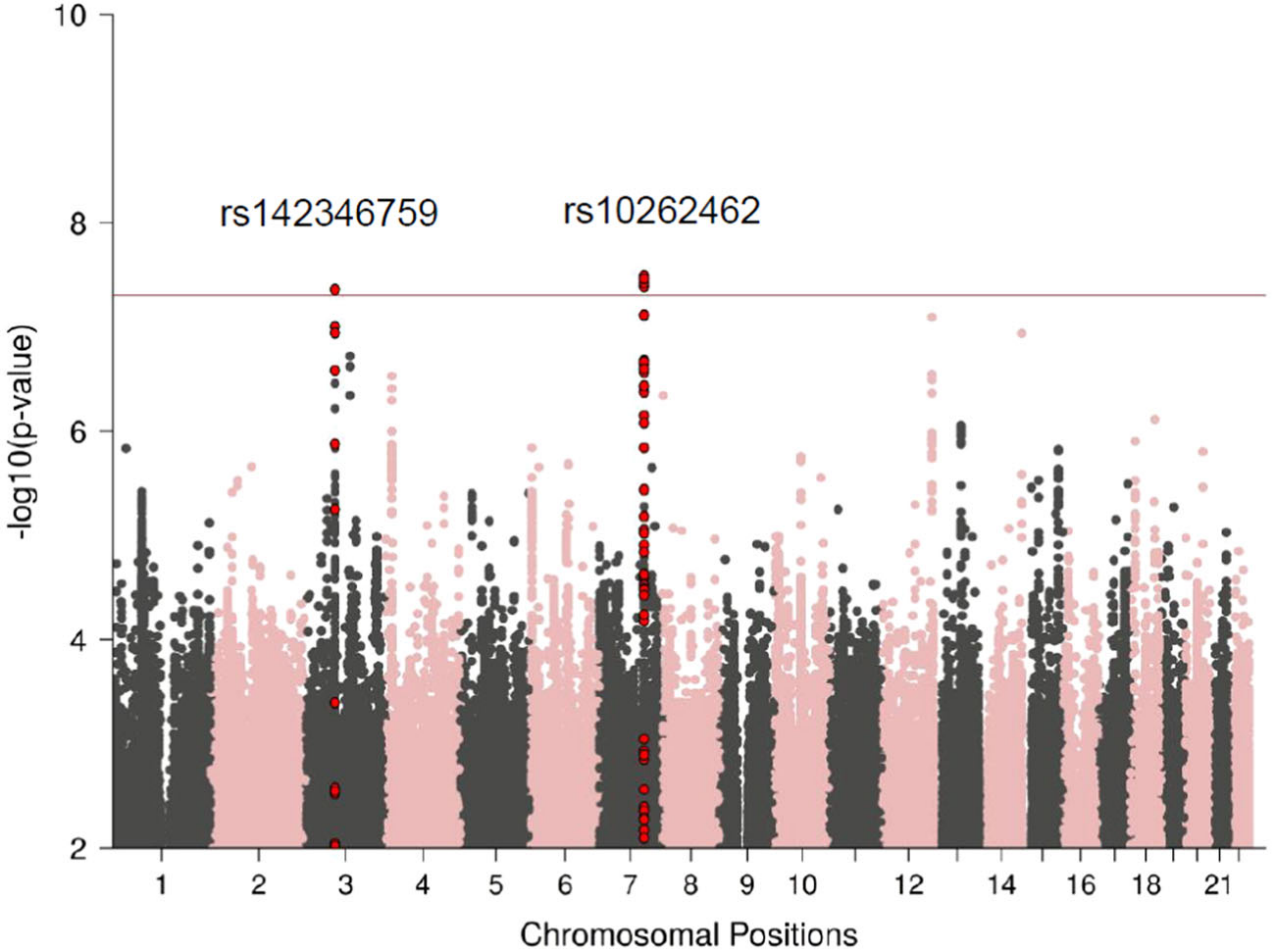
Manhattan plot of UKBB GWAS for childhood maltreatment, showing the top variants. The horizontal line represents genome-wide significance at *p* < 5 × 10^−8^.

We report GWAS results from our discovery dataset (UKBB) (*n* = 124,711) and meta-analysis (*n* = 151,001). In our UKBB discovery dataset, we identified two genome-wide significant loci (*p* < 5 × 10^−8^) associated with childhood maltreatment (Table 1 and Fig. 1), rs142346759 (chr3, beta = 0.015, *p* = 4.35 × 10^−8^) and rs10262462 (chr7, beta = −0.016, *p* = 3.24 × 10^−8^). These variants remained significant in the meta-analysis (Table 1, Supplementary Figs. 1 and 2). Additional variants on chromosome 7 (rs1859100, beta = 0.015, *p* = 3.91 × 10^−8^) and chromosome 12 (rs917577, beta = 0.017, *p* = 2.64 × 10^−8^) (Supplementary Fig. 3), also achieved genome-wide significance in the meta-analysis. Running an ordinal regression on these hits in the UKBB led to similar results (data not shown). None of these hits were replicated in PGC1.5 (Table 1 and Supplementary Fig. 4).

**Table 1:** Genome-wide significant hits in the UK Biobank, PGC-PTSD Freeze 1.5 (PGC 1.5), and meta-analyses.

Variant	Chr	Position (bp)	Gene	A1	A2	A1 freq	Discovery: UK Biobank				Replication: Freeze 1.5						Meta-analysis					
							*Z*-score	Beta	*p*-Value	*n*	*Z*-score	Beta^a^	*P*-value	*I*² (%)	P-het	*n*	*Z*-score	Beta^a^	*p*-Value	*I*² (%)	P-het	*n*
rs142346759	3	71,362,232	*FOXP1*	G	A	0.039	5.476	0.015	4.35E−08	124,711	1.490	0.016	0.136	5	0.394	10,858^b^	5.674	0.033	1.40E−08	0	0.491	135,569^b^
rs1859100	7	114,194,615	*FOXP2*	G	T	0.597	5.517	0.016	3.45E−08	124,711	0.846	0.007	0.397	0	0.978	14,495	5.495	0.015	3.91E−08	0	0.973	139,206
rs10262462	7	114,180,062	*FOXP2*	A	G	0.403	−5.528	−0.016	3.24E−08	124,711	−0.729	−0.006	0.466	0	0.969	14,495	−5.468	−0.015	4.56E−08	0	0.958	139,206
rs917577	12	126,548,817	Intergenic	C	G	0.265	5.367	0.015	8.02E−08	124,711	1.476	0.016	0.14	0	0.781	11,463	5.564	0.017	2.64E−08	0	0.831	136,174

^a^An approximation was used to transform the *Z*-statistics from the effective sample-size-weighted meta-analysis (the output of the software METAL) into a beta value^74^. This was calculated on the scale of the MRS dataset

^b^As this variant has a MAF of < 5% in the UKBB, only studies with a minor allele count of at least five alleles were included in the meta analysis

Quantile–quantile (qq) plots indicate minimal inflation of *p*-values across studies (Supplementary Figs. 5–7). Using the LDSR intercept method, polygenic effects account for 93% and 94% of the observed inflation in test statistics for the UKBB dataset (intercept = 1.0096, SE = 0.0064) and meta-analysis (intercept = 1.0095, SE = 0.0077), respectively (Supplementary Figs. 5 and 7), consistent with minimal population stratification and cryptic relatedness.

### Integration with functional genomic data

Using the web-based tool FUMA, the two UKBB GWAS hits were each annotated to two genes, *FOXP1* and *FOXP2* (Table 2). Gene-based analysis of the UKBB GWAS summary statistics further identified three gene-wide significant genes, *KIF26B* (*p* = 1.67 × 10^−7^), *CNTNAP5* (*p* = 8.89 × 10^−7^), and *EXOC2* (*p* = 2.04 × 10^−6^) from a total of 18,989 protein-coding genes. Gene-set analysis did not reveal any significant pathways associated with childhood maltreatment. Limited functionality of the two risk variants (rs142346759 and rs10262462) was observed (Table 2). One of the SNPs in LD for the risk variant on chromosome 3, rs142346759, obtained a CADD score of >12.37, indicating that this SNP may be deleterious. Six of the SNPs in LD with the risk variant on chromosome 7, rs10262462, had a CADD score of >12.37. No significant eQTLs were identified for either risk locus.

**Table 2:** Functional mapping and annotation of UKBB GWAS and meta-analysis.

Sample	GWAS hit lead variant	GWAS *p*-value	Position (hg19)	#SNPs in LD (*r*^2^ > 0.6)	Genomic coordinated risk locus (hg19)	Predicted genes in risk locus	SNPs in LD with CADD scores > 12.37	SNPs in LD with RegulomeDB scores < 5	Chromatin state analysis (Roadmap Epigenomics) in neuronal cell lines/tissues^a^	eQTL (28 neuronal tissue/cell lines from CommonMind Consortium, BRAINEAC or GTEx v7)
UKBB GWAS	rs142346759	4.35E−08	3: 71362232	5	chr3:71355932–71417539	*FOXP1* intronic	rs34936081	rs13083684 (intronic)	Mainly quiecent	None
	rs10262462	4.36E−08	7: 114180062	77	chr7:114015707–114287116	*FOXP2* intronic	rs7785701,rs6466488,rs10249234,rs9332390,rs66823671,rs12533005	rs2396753 (intronic), rs12536335 (intronic)	Mainly quiecent	None
Meta-analysis	rs1859100	3.91E−08	7: 114194615	74	chr7:114015707–114287116	*FOXP2* intronic	rs66823671,rs71149745,rs7785701,rs12533005,rs9332390,rs10249234, rs1476535, rs6466488	rs2396753 (intronic), rs12536335 (intronic), rs9332390 (intronic)	Mainly quiecent	None
	rs917577	2.64E−08	12:126548817	27	chr12:126548816–126585545	Intergenic	None	None	Mainly quiecent	None

^a^In neuronal cell lines/tissues E053, E054, E067, E068, E069, E070, E071, E072, E073, E074, E081, E082, E125

The chromosome 7 variant identified in the meta-analysis, rs1859100, also mapped to the gene *FOXP2* and is located in the same genomic risk locus (chr7:114,015,707–114,287,116 base pairs) as rs10262462. The other hit observed in the meta-analysis, rs917577, was mapped to an intergenic region on chromosome 12. This variant obtained an RDB categorical score of 2B, indicating that it is likely to affect transcription factor binding. No eQTLs exist in the selected tissue types for this region (Table 2).

### Heritability of reported childhood maltreatment

GWAS summary statistics were used to estimate the *h*^2^_snp_ of childhood maltreatment with the tool LDSR (Table 3). The *h*^2^_snp_ was estimated at 0.057 (*p* = 1.60 × 10^−32^) for the UKBB discovery dataset and 0.123 (*p* = 0.002) for PGC1.5. The *h*^2^_snp_ for the meta-analysis was 0.057 (*p* = 4.48 × 10^−46^).

**Table 3:** Heritability estimates based on LD-score regression (LDSR).

Sample	*n*	*h*^2^_snp_	SE	*Z*	*p*-value
UKBB	124,711	0.057	0.005	11.40	1.60E−32
PGC1.5	26,290	0.123	0.040	3.08	2.00E−03
Meta-analysis	151,001	0.057	0.004	14.25	4.48E−46

Estimates are calculated for the UK biobank (UKBB), the PGC-PTSD Freeze 1.5 (PGC1.5), and meta-analysis

### Polygenic risk scoring

We assessed the predictive value of PRS for childhood maltreatment, using our largest cohort, the UKBB, as a training sample. Our analyses showed a highly significant increase in effect size to develop childhood maltreatment across PRS quantiles in the PGC1.5 target sample, with a variance explained of *r*^2^ = 0.0025 (*p* = 1.8 × 10^−15^). Participants in the 5th quantile of genetic risk had significantly higher childhood maltreatment scores than subjects in the 1st quantile (beta = 0.042, *p* = 4.78 × 10^−16^; Supplementary Fig. 8). Since women reported significantly more childhood maltreatment than men (PGC1.5 mean childhood maltreatment: women = 0.32, men = 0.127, *p* < 1 × 10^−80^), PRS were also calculated separately for women and men. When stratified by sex, PRS had significantly higher explanatory power in women (*r*^2^ = 0.0053) relative to men (*r*^2^ = 0.0015) (*p* = 0.0002, Supplementary Fig. 8).

### Genetic correlations of reported childhood maltreatment with other traits and disorders

**Fig. 2:**
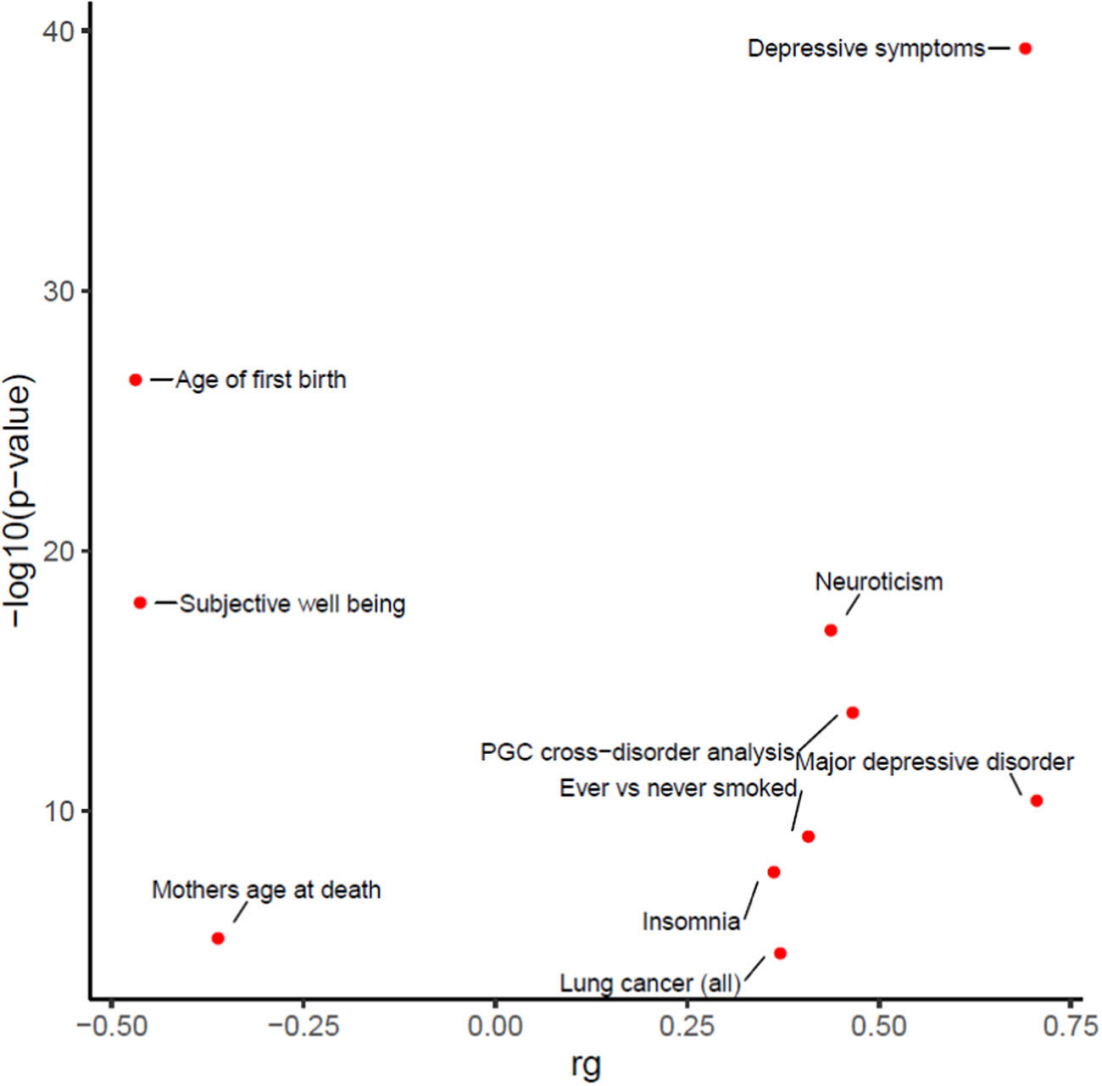
Top ten genetic correlations between several groups of traits (from psychiatric, anthropomorphic, smoking behavior, reproductive, aging, education, autoimmune, and cardio-metabolic categories) and childhood maltreatment (meta-analysis).

All pairwise genetic correlations are listed in Supplementary Table 3. The *r*_g_ for childhood maltreatment between the UKBB and PGC1.5 datasets was 0.63 (*p* = 3.28 × 10^−6^). To determine whether there is significant genetic overlap between childhood maltreatment and other traits and disorders, pairwise genetic correlations were calculated using the web-based tool LD Hub. A total of 27 significant correlations (Bonferroni-corrected *p*-value threshold = 0.05/247 = 0.0002) were found between childhood maltreatment in the meta-analysis and 247 non-UKBB traits. The top 10 highest genetic correlations are plotted in Fig. 2 with depressive symptoms (*r*_g_ = 0.70, *p* = 4.65 × 10^−40^) having the most significant correlation with childhood maltreatment. There were also positive genetic correlations with “MDD” (*r*_g_ = 0.71, *p* = 4.13 × 10^−11^), “PGC cross-disorder analysis” (*r*_g_ = 0.47, *p* = 1.62 × 10^−14^) and “neuroticism” (*r*_g_ = 0.44, *p* = 1.14 × 10^−17^). Significant negative genetic correlations between childhood maltreatment and “age of first birth” (*r*_g_ = −0.47, *p* = 2.61 × 10^−27^), “subjective well-being” (*r*_g_ = −0.46, *p* = 1.00 × 10^−18^), and “mother’s age at death” (*r*_g_ = −0.36, *p* = 7.42 × 10^−6^) were also observed.

### Conditional analyses of childhood maltreatment top hits

As depressive symptoms (*r*_g_ = 0.70, *p* = 4.65 × 10^−40^) and MDD (*r*_g_ = 0.71, *p* = 4.13 × 10^−11^) were the most genetically correlated with childhood maltreatment, we conditioned the top hits from our meta-analysis for the effects of MDD using publicly available summary statistics for MDD^42^. We found that effect sizes for the four top hits for childhood maltreatment remained similar when adjusted for the effects of MDD (Supplementary Table 4). These findings indicate that the genetic variants identified here are specific to childhood maltreatment when tested in the context of MDD, the disorder genetically most significantly correlated with childhood maltreatment.

## Discussion

The main findings of this study were that (1) variants located in the genes *FOXP1* and *FOXP2* and on chromosome 12 are significantly associated with childhood maltreatment, (2) the SNP-based estimate of childhood maltreatment is ~6%, (3) PRS of self-reported childhood maltreatment derived from a discovery cohort can significantly predict this phenotype in a target cohort, with 0.25% of variance explained, and (4) childhood maltreatment is significantly genetically correlated with “depressive symptoms” and “MDD”, “neuroticism”, “age of first birth”, and “subjective well-being”, despite showing evidence that our top hits may be specific to childhood maltreatment when conditioning on MDD.

Two genome-wide loci for childhood maltreatment identified in our discovery dataset were also significant in the meta-analysis: rs142346759 (chr3p13), an intronic variant in *FOXP1* and rs10262462 (chr7q31.1) an intronic variant located in *FOXP2*. Both genes form part of the forkhead box superfamily of transcription factors which are widely expressed, and which play important roles during development and adulthood. *FOXP1* and *FOXP2* fall under the FOXP sub-family (also comprising *FOXP3* and *FOXP4*) which has functions in oncogenic and tumor suppressive pathways^43^. *FOXP2* contains highly conserved genomic sites, including an intronic region within this gene, located about 107 kb downstream from our risk variant^44^. FOXP1 and FOXP2 have ~60% homology at the amino acid level (https://www.ncbi.nlm.nih.gov/books/NBK7023/) and both proteins have been implicated in cognitive disorders, including expressive language impairment^45^. In the meta-analysis, we observed an additional genome-wide variant, located in an intergenic region on chromosome 12, but as this variant does not map to a particular gene, its possible biological mechanism is unclear.

Notably, variation within *FOXP1* has been found to have associations with language impairment, internalizing symptoms, and externalizing symptoms^46^. *FOXP2* has mainly been investigated in regards to speech and language development^47^, but has also been found to be associated with depression^48^ and attention deficit hyperactivity disorder (ADHD)^49^. Further, an intronic variant in the *FOXP2* gene, rs727644, has been associated with risk-taking behavior^50,51^. While most work on childhood maltreatment has emphasized subsequent risk for mental and physical disorders, it is possible that externalizing behaviors increase risk for childhood trauma^52^, consistent with a non-passive rGE mechanism. Alternatively, phenotypes such as depression or neuroticism may increase the likelihood of individuals recalling childhood maltreatment^53,54^.

In this study we estimated SNP-based heritability for childhood maltreatment to be ~6%. A first possibility, in line with a link between *FOXP* variants and externalizing symptoms, is that genetic factors influence environmental factors indirectly through temperament and behavior^9^. A second possibility, consistent with the link of *FOXP* variants to internalizing symptoms and depression, is that genetic factors influence the recall of childhood maltreatment. In particular, retrospective assessment of childhood maltreatment may be limited by recall bias and the respondent’s subjective assessment of the event^55,56^. Indeed, a recent systematic review found very low concordance between prospective and retrospective measures of childhood maltreatment^57^ and those who retrospectively report childhood adversity were at greater risk for having psychopathology than those who prospectively reported childhood maltreatment^58^.

A twin-based study estimated the heritability of reported childhood maltreatment (comprising physical, and sexual maltreatment and neglect) to be 6%^7^, the same as our SNP-based estimate. As twin-based studies capture latent heritability across the entire genome, these heritability estimates are generally higher than SNP-based heritability estimates, which are limited to common variation and by the number of markers present and tagged on the genotyping array used^15^. However, in this twin study, when considering each maltreatment category separately, the heritability of childhood physical maltreatment, sexual maltreatment, and neglect was 28%, 0%, and 24%, respectively. This suggests that only physical abuse and neglect are heritable and that sexual abuse is not genetically influenced. It is notable that these twin data, then, do not support an rGE for some abuse types (i.e. sexual abuse).

We show that PRS derived from the UKBB was significantly predictive of childhood maltreatment in PGC1.5, explaining 0.25% of the variance for this exposure. Although the variance explained is relatively modest, we expect greater prediction accuracy with future larger sample sizes. When stratifying by sex, PRS had significantly higher explanatory power in women relative to men. This is expected as women had higher mean self-reported childhood maltreatment scores than men in PGC1.5.

The PRS results suggests a polygenic architecture for self-reported childhood maltreatment but does not indicate the mechanism by which genetic factors are able to influence this exposure. However, our finding of positive genetic correlations between childhood maltreatment, depressive symptoms, and MDD provides support for the hypothesis that genetic factors predisposing to reporting early life maltreatment overlap with those underlying depression. Genetic correlations between depression, stressful life events, and lifetime trauma have led to the hypothesis that genes increasing risk for the development of depression predispose individuals to entering into adverse environments^59,60^. Depressed individuals with and without trauma exposure differ in associated genetic variation, with trauma-exposed individuals having greater SNP-based heritability, supporting this hypothesis^26,61^. On the other hand, polygenic scores for MDD were associated with greater reporting of stressful life events in individuals with MDD^62^. Indeed, current mood can influence the recall of childhood experiences, and individuals with current depression are at an increased likelihood of reporting early life adversity^63^. Notably, although we show that childhood maltreatment is significantly genetically correlated with depression, results from our conditional GWAS analysis indicates that our top four hits are specific to self-reported childhood maltreatment, favoring a non-passive rGE mechanism for childhood maltreatment.

In addition to depression, we found significant positive genetic correlations between childhood maltreatment and “neuroticism” and “PGC cross-disorder analysis” (comprised of GWAS summary statistics of five psychiatric disorders: autism spectrum disorder, attention deficit-hyperactivity disorder, bipolar disorder, MDD, and schizophrenia). We observed negative genetic correlations of childhood maltreatment with “age of first birth” and “subjective well-being”. Associations between early life maltreatment and each of these phenotypes have previously been observed^61,64–72^. Further investigation is required to delineate the mechanisms that play a role in the relationship between childhood maltreatment and these outcomes.

Our study had a number of limitations that deserve emphasis. First, the genetic correlation between the UKBB and PGC1.5 datasets was only 0.63, indicating differences between the datasets, which possibly explains the non-replication of our top hit and of greater SNP heritability in PGC1.5. The UKBB dataset comprises healthy volunteers who are typically of a higher socioeconomic status and in better overall health than the general population of comparable age^73^, and the findings reported here may not be generalizable to the general population. However, it is also worth noting that the top hits were significant in the meta-analysis, where additional hits for childhood maltreatment were detected in an intergenic region on chromosome 12. Second, although many of the study sites included in the final meta-analysis utilized the well-validated CTQ, childhood maltreatment was measured in a diversity of ways across the different studies. Thus, our main phenotype was not homogenous and may reflect different aspects of childhood maltreatment in different contributing studies.

This is the first large-scale genetic study to identify specific variants associated with self-reported childhood maltreatment. Variation in *FOXP* genes and the polygenic architecture associated with childhood maltreatment may put individuals at greater risk for maltreatment. Alternatively, however, these variants may be associated with a greater likelihood of reporting maltreatment, given the high genetic correlation between childhood maltreatment and depression, and neuroticism. Using the available data, we are unable to indicate definitively which of these explanations is a better one, and it is possible that different mechanisms have more robust explanatory power in accounting for different abuse subtypes as well as different associated psychopathologies. A clearer understanding of the genetic relationships of childhood maltreatment, including particular abuse subtypes, with a range of different phenotypes, may ultimately be useful in developing targeted treatment and prevention strategies.

## Disclaimer

The views, opinions, and/or findings contained in this report are those of the authors and should not be construed as official Department of the Army position, policy, or decision, unless so designated by other official documentation. Citations of commercial organizations or trade names in this report do not constitute an official Department of the Army endorsement or approval of the products or services of these organizations.

## Supplementary information

Supplementary Table 1

Supplementary Table 2

Supplementary Table 3

Supplementary Table 4

Supplementary Figures

Supplementary Note

## Data Availability

The full meta-analysis summary statistics are available for download from the Psychiatric Genomics Consortium at https://www.med.unc.edu/pgc/results-and-downloads/. Access to individual-level data for available datasets may be requested through the PGC Data Access Portal at https://www.med.unc.edu/pgc/shared-methods/data-access-portal/. All other data that support the findings of this study are available from the corresponding author upon request.

## Code availability

GWAS and associated scripts are available at https://github.com/nievergeltlab/ct_gwas/.
