# Supplementary material for: Genomic influences on self-reported childhood maltreatment
Source: Transl Psychiatry. 2020 Jan 27;10:38. doi: 10.1038/s41398-020-0706-0 (PMC7026037; doi:10.1038/s41398-020-0706-0)
Supplement: Supplementary file 1 — Supplementary Table 1 [file 41398_2020_706_MOESM1_ESM.docx]

| **Supplementary Table 1: Demographics of PGC-PTSD and UKBB cohorts with childhood maltreatment** | | | | |  |  |  |  |  |  |
| --- | --- | --- | --- | --- | --- | --- | --- | --- | --- | --- |
|  |  |  |  |  |  |  |  |  |  |  |
| **Study** | **Study** | **PGC** |  |  |  |  |  |  |  |  |
| **number¹** | **abbreviation¹** | **data sharing** | **Genotyping array** | **Childhood maltreatment measure** | **N** | **Score range** | **Score mean (SE)** | **Sex (% male)** | **Mean age (SD)** | **Neff²** |
| 1 | MRSC | genotypes | OmniExpressExome8 + Custom | Childhood Trauma Questionnaire (CTQ) | 2258 | 0-3 | 0.43 (0.01) | 100% | 23.3 (3.2) | 565 |
| 2 | ONGA | genotypes | PsychArray | abbreviated CTQ | 212 | 0-3 | 0.53 (0.06) | 77.3% | 33.1 (10.2) | 91 |
| 5 | NHS2 | genotypes | PsychArray | CTQ, Conflict Tactics Scale (CTS) , Sexual Experiences Survey | 1331 | 1-3 | 1.81 (0.02) | 0% | 35.8 (4.3) | 1217 |
| 6 | GSDC | genotypes | Omni1-Quad | Semi-Structured Assessment for Drug Dependence and Alcoholism | 1315 | 0-4 | 0.53 (0.03) | 57.7% | 38.2 (10.9) | 420 |
| 10 | BRYA | genotypes | PsychArray | Early life stress questionnaire | 315 | 0-4 | 0.45 (0.05) | 73.7% | 47.2 (13.7) | 49 |
| 13 | NHRV | genotypes | PsychArray | Trauma History Screen | 1891 | 0-2 | 0.26 (0.01) | 90.8% | 63.7 (13.0) | 368 |
| 14 | NSS1 | summary data | OmniExpressExome8 + Custom | Self-administered questionnaire | 7995 | 0-3 | 0.48 (0.009) | 82.7% | 21.0 (3.3) | 3856 |
| 15 | NSS2 | summary data | PsychArray | Self-administered questionnaire | 2833 | 0-3 | 0.67 (0.02) | 79.1% | 20.3 (3.1) | 1897 |
| 16 | PPDS | summary data | OmniExpressExome8 + Custom | Self-administered questionnaire | 7853 | 0-3 | 0.28 (0.008) | 93.7% | 26.0 (6.0) | 2199 |
| 17 | KSUD | genotypes | PsychArray 1.1 | CTQ | 220 | 0-5 | 2.21 (0.11) | 60% | 34.9 (11.0) | 193 |
| 18 | BOBA | genotypes | PsychArray 1.1 | Measure created for use in this study. | 138 | 0-5 | 1.17 (0.09) | 35.5% | 14.7 (1.7) | 39 |
| 21 | GUTS | genotypes | PsychArray 1.1 | Gallup Poll, CTS | 515 | 0-5 | 0.90 (0.05) | 26.6% | 26.2 (1.7) | 157 |
| 22 | NHSY | genotypes | PsychArray 1.1 | Gallup Poll, CTS | 5408 | 0-6 | 2.00 (0.02) | 0% | 51.7 (4.3) | 3032 |
| 36 | BRY2 | genotypes | PsychArray 1.1 | Early life stress questionnaire | 121 | 0-6 | 2.07 (0.19) | 43.1% | 41.1 (12.5) | 92 |
| 37 | FEEN | genotypes | PsychArray 1.1 | Standardized Trauma Interview | 88 | 0-2 | 0.73 (0.08) | 18.6% | 37.3 (12.1) | 84 |
| 39 | TEIC | genotypes | PsychArray 1.1 | CTQ | 42 | 0-5 | 3.05 (0.32) | 0% | 37.5 (12.9) | 18 |
| 40 | NUIT | genotypes | PsychArray 1.1 | Traumatic Life Events Questionnaire and Briere | 88 | 1-3 | 1.38 (0.07) | 0% | 19.2 (1.1) | 39 |
| 52 | FTCB | genotypes | PsychArray | CTQ | 858 | 0-3 | 0.29 (0.02) | 95.4% | 27.1 (5.9) | 179 |
| 60 | UKBB | summary data | Affymetrix UK Biobank Axiom array | summary data | 124 711 | 0-3 | summary data | summary data | summary data | 25 742 |
|  |  |  |  |  |  |  |  |  |  |  |
| **Key** |  |  |  |  |  |  |  |  |  |  |
| MRSC | Marine Resiliency Study | |  |  |  |  |  |  |  |  |
| ONGA | Ohio National Guard | |  |  |  |  |  |  |  |  |
| NHS2 | Nurses Health Study II | |  |  |  |  |  |  |  |  |
| GSDC | Yale-Penn Study | |  |  |  |  |  |  |  |  |
| BRYA | Ash Wednesday and IVS | |  |  |  |  |  |  |  |  |
| NHRV | National Health and Resilience in Veterans Study | | |  |  |  |  |  |  |  |
| NSS1 | Army Study to Assess Risk and Resilience in Service members | | |  |  |  |  |  |  |  |
| NSS2 | Army Study to Assess Risk and Resilience in Service members | | |  |  |  |  |  |  |  |
| PPDS | Army Study to Assess Risk and Resilience in Service members | | |  |  |  |  |  |  |  |
| KSUD | Genetics of Posttraumatic Stress Disorder/Substance Use Disorder Comorbidity | | | |  |  |  |  |  |  |
| BOBA | Bounce Back Now | |  |  |  |  |  |  |  |  |
| GUTS | Growing Up Today Study | |  |  |  |  |  |  |  |  |
| NHSY | Nurses Health Study II | |  |  |  |  |  |  |  |  |
| BRY2 | Sydney Neuroimaging | |  |  |  |  |  |  |  |  |
| FEEN | OPT and CHOICE | |  |  |  |  |  |  |  |  |
| TEIC | McLean Trauma Sample | |  |  |  |  |  |  |  |  |
| NUIT | NIU Trauma Orcutt | |  |  |  |  |  |  |  |  |
| FTCB | Fort Campbell study | |  |  |  |  |  |  |  |  |
| UKBB | UK Biobank |  |  |  |  |  |  |  |  |  |
|  |  |  |  |  |  |  |  |  |  |  |
| ¹Study number and abbreviation correspond to the PGC-PTSD freeze 2 (https://www.biorxiv.org/content/10.1101/458562v1) | | | | |  |  |  |  |  |  |
| ²Neff: Effective sample size; see Methods for details | | | |  |  |  |  |  |  |  |
|  |  |  |  |  |  |  |  |  |  |  |
