# Supplementary material for: Genomic influences on self-reported childhood maltreatment
Source: Transl Psychiatry. 2020 Jan 27;10:38. doi: 10.1038/s41398-020-0706-0 (PMC7026037; doi:10.1038/s41398-020-0706-0)
Supplement: Supplementary file 2 — Supplementary Table 2 [file 41398_2020_706_MOESM2_ESM.docx]

| **Supplementary Table 2: Child Trauma Questionnaire (CTQ) abuse category score thresholds** | | | | |
| --- | --- | --- | --- | --- |
|  |  |  |  |  |
|  | **Classification** | | | |
| **Category** | **None or Minimal** | **Low to Moderate** | **Moderate to Severe¹** | **Severe to Extreme¹** |
| Emotional Abuse | 5-8 | 9-12 | 13-15 | ≥16 |
| Physical Abuse | 5-7 | 8-9 | 10-12 | ≥13 |
| Sexual Abuse | 5 | 6-7 | 8-12 | ≥13 |
|  |  |  |  |  |
| ¹Participants scoring in this range are given a count score of 1 for that respective abuse category. | | | | |
| The childhood maltreatment count score is the sum of the three abuse categories^20^. | | | | |
