# Supplementary material for: Genomic influences on self-reported childhood maltreatment
Source: Transl Psychiatry. 2020 Jan 27;10:38. doi: 10.1038/s41398-020-0706-0 (PMC7026037; doi:10.1038/s41398-020-0706-0)
Supplement: Supplementary file 3 — Supplementary Table 3 [file 41398_2020_706_MOESM3_ESM.docx]

| **Supplementary Table 3: Genetic correlations (bivariate LD score regression) of childhood maltreatment with several traits and disorders for data publicly available on LD Hub.** | | | | | | | | | | | | | | | | | | | |
| --- | --- | --- | --- | --- | --- | --- | --- | --- | --- | --- | --- | --- | --- | --- | --- | --- | --- | --- | --- |
| Significant correlations with the childhood maltreatment meta-analysis after Bonferroni-correction for 247 comparisons are highlighted in blue (p ≤ 2.00E-04). | | | | | | | | | | |  |  |  |  |  |  |  |  |  |
|  |  |  | |  |  |  |  |  |  |  | | |  |  |  |  |  |  |  |
|  |  |  | |  |  |  |  |  |  | **Trait 2** | | | | | |  |  |  | |
| **Trait1** | **Trait2** | **PMID** | | **Category** | **Ethnicity** | **rg** | **se** | **z** | **p** | **h2_obs** | | | **h2_obs_se** | **h2_int** | **h2_int_se** | **gcov_int** | **gcov_int_se** | **note** | |
| childhood maltreatment | Waist circumference | 25673412 | | anthropometric | Eur | 0.2266 | 0.0395 | 5.7369 | **9.6419e-09** | 0.1217 | | | 0.0053 | 0.8488 | 0.0087 | 0.0044 | 0.0059 |  | |
| childhood maltreatment | Ever vs never smoked | 20418890 | | smoking_behaviour | Eur | 0.4077 | 0.0666 | 6.1221 | **9.2379e-10** | 0.072 | | | 0.007 | 1.0039 | 0.0069 | 0.0003 | 0.0051 |  | |
| childhood maltreatment | HDL cholesterol | 20686565 | | lipids | Eur | -0.1961 | 0.0498 | -3.9344 | **8.3406e-05** | 0.1184 | | | 0.028 | 1.0832 | 0.0617 | 0.0001 | 0.0065 |  | |
| childhood maltreatment | Number of children ever born | 27798627 | | reproductive | Eur | 0.3455 | 0.0562 | 6.144 | **8.0476e-10** | 0.0253 | | | 0.002 | 0.9758 | 0.0083 | 0.0037 | 0.0052 |  | |
| childhood maltreatment | Lung cancer | 27488534 | | cancer | Eur | 0.3089 | 0.078 | 3.9604 | **7.4820e-05** | 0.3091 | | | 0.0721 | 1.0196 | 0.0089 | -0.0029 | 0.0057 |  | |
| childhood maltreatment | Mothers age at death | 27015805 | | aging | Eur | -0.3612 | 0.0806 | -4.4813 | **7.4200e-06** | 0.0379 | | | 0.008 | 1.0098 | 0.0085 | -0.0142 | 0.0052 |  | |
| childhood maltreatment | Obesity class 1 | 23563607 | | anthropometric | Eur | 0.2069 | 0.0384 | 5.3884 | **7.1080e-08** | 0.2169 | | | 0.0118 | 1.0195 | 0.0115 | 0.0022 | 0.0064 |  | |
| childhood maltreatment | Depressive symptoms | 27089181 | | psychiatric | Eur | 0.6905 | 0.0521 | 13.2478 | **4.6478e-40** | 0.0482 | | | 0.0037 | 1 | 0.0073 | 0.0189 | 0.0056 |  | |
| childhood maltreatment | Major depressive disorder | 22472876 | | psychiatric | Eur | 0.7056 | 0.1069 | 6.5992 | **4.1328e-11** | 0.1424 | | | 0.0264 | 1.0189 | 0.0071 | -0.0038 | 0.0057 |  | |
| childhood maltreatment | Lung cancer (all) | 24880342 | | cancer | Eur | 0.3715 | 0.0891 | 4.1706 | **3.0386e-05** | 0.1281 | | | 0.031 | 1.0105 | 0.0092 | -0.0053 | 0.0059 |  | |
| childhood maltreatment | Years of schooling (proxy cognitive performance) | 25201988 | | education | Eur | -0.2338 | 0.05 | -4.6743 | **2.9497e-06** | 0.1091 | | | 0.0078 | 1.0263 | 0.0101 | -0.0066 | 0.0061 |  | |
| childhood maltreatment | Years of schooling 2016 | 27225129 | | education | Eur | -0.2322 | 0.0332 | -6.9886 | **2.7760e-12** | 0.1277 | | | 0.0049 | 0.9384 | 0.0124 | -0.0218 | 0.0064 |  | |
| childhood maltreatment | Age of first birth | 27798627 | | reproductive | Eur | -0.4689 | 0.0433 | -10.8255 | **2.6060e-27** | 0.0638 | | | 0.0038 | 0.9524 | 0.009 | -0.017 | 0.0063 |  | |
| childhood maltreatment | Obesity class 2 | 23563607 | | anthropometric | Eur | 0.2647 | 0.0444 | 5.9645 | **2.4537e-09** | 0.1845 | | | 0.0123 | 1.002 | 0.0099 | -0.0019 | 0.0066 |  | |
| childhood maltreatment | Insomnia | 28604731 | | sleeping | Eur | 0.3628 | 0.065 | 5.5853 | **2.3323e-08** | 0.0487 | | | 0.0051 | 1.0045 | 0.0082 | 0.0179 | 0.0054 |  | |
| childhood maltreatment | Body mass index | 20935630 | | anthropometric | Eur | 0.2343 | 0.037 | 6.3386 | **2.3185e-10** | 0.1912 | | | 0.0098 | 1.0144 | 0.0123 | -0.0057 | 0.0058 |  | |
| childhood maltreatment | Obesity class 3 | 23563607 | | anthropometric | Eur | 0.2897 | 0.0684 | 4.233 | **2.3060e-05** | 0.1217 | | | 0.0142 | 0.98 | 0.0092 | -0.0026 | 0.0068 |  | |
| childhood maltreatment | Waist-to-hip ratio | 25673412 | | anthropometric | Eur | 0.1856 | 0.0391 | 4.7507 | **2.0275e-06** | 0.1136 | | | 0.0073 | 0.9242 | 0.0102 | 0.0086 | 0.0061 |  | |
| childhood maltreatment | Extreme bmi | 23563607 | | anthropometric | Eur | 0.2254 | 0.0529 | 4.2632 | **2.0156e-05** | 0.6922 | | | 0.0547 | 1.03 | 0.0113 | -0.0041 | 0.0077 |  | |
| childhood maltreatment | PGC cross-disorder analysis | 23453885 | | psychiatric | Eur | 0.4661 | 0.0607 | 7.678 | **1.6158e-14** | 0.1655 | | | 0.0138 | 1.0285 | 0.0125 | -0.0039 | 0.0082 |  | |
| childhood maltreatment | Insomnia | 27992416 | | sleeping | Eur | 0.3043 | 0.0579 | 5.2549 | **1.4809e-07** | 0.1357 | | | 0.0122 | 1.0031 | 0.0088 | 0.014 | 0.0058 |  | |
| childhood maltreatment | Overweight | 23563607 | | anthropometric | Eur | 0.1922 | 0.0443 | 4.339 | **1.4316e-05** | 0.1103 | | | 0.0068 | 1.027 | 0.0109 | 0.0035 | 0.0059 |  | |
| childhood maltreatment | Hip circumference | 25673412 | | anthropometric | Eur | 0.1713 | 0.0391 | 4.3811 | **1.1806e-05** | 0.1286 | | | 0.0059 | 0.863 | 0.0093 | 0.0004 | 0.0057 |  | |
| childhood maltreatment | Neuroticism | 27089181 | | personality | Eur | 0.4369 | 0.0511 | 8.5591 | **1.1378e-17** | 0.0895 | | | 0.0079 | 0.9935 | 0.013 | 0.0251 | 0.0061 |  | |
| childhood maltreatment | College completion | 23722424 | | education | Eur | -0.2628 | 0.0538 | -4.8838 | **1.0408e-06** | 0.0813 | | | 0.006 | 1.0209 | 0.0093 | -0.003 | 0.006 |  | |
| childhood maltreatment | Subjective well being | 27089181 | | psychiatric | Eur | -0.4629 | 0.0524 | -8.835 | **1.0010e-18** | 0.0261 | | | 0.0021 | 0.9966 | 0.0075 | -0.0106 | 0.0051 |  | |
| childhood maltreatment | Age at Menarche | 25231870 | | reproductive | Eur | -0.1274 | 0.0334 | -3.8168 | **0.0001** | 0.2074 | | | 0.0107 | 0.9504 | 0.0133 | 0.0012 | 0.0067 |  | |
| childhood maltreatment | Fathers age at death | 27015805 | | aging | Eur | -0.2878 | 0.0768 | -3.746 | 0.0002 | 0.0439 | | | 0.0072 | 1.0133 | 0.0075 | -0.016 | 0.0052 |  | |
| childhood maltreatment | Glutamine | 27005778 | | metabolites | Eur | -0.0029 | 0.1119 | -0.0261 | 0.9792 | 0.0613 | | | 0.0215 | 1.0197 | 0.0085 | -0.0044 | 0.0047 |  | |
| childhood maltreatment | LDL cholesterol | 20686565 | | lipids | Eur | 0.0019 | 0.0554 | 0.0344 | 0.9725 | 0.1024 | | | 0.0292 | 1.0609 | 0.0515 | 0.0041 | 0.0067 |  | |
| childhood maltreatment | Mean Thalamus | 25607358 | | brain_volume | Eur | -0.0039 | 0.1101 | -0.0358 | 0.9715 | 0.1298 | | | 0.038 | 0.984 | 0.0076 | -0.0056 | 0.0054 |  | |
| childhood maltreatment | Total cholesterol in HDL | 27005778 | | metabolites | Eur | 0.0045 | 0.102 | 0.0437 | 0.9652 | 0.0889 | | | 0.0311 | 1.0096 | 0.0152 | -0.0072 | 0.0054 |  | |
| childhood maltreatment | Glucose | 27005778 | | metabolites | Eur | -0.006 | 0.0839 | -0.072 | 0.9426 | 0.0909 | | | 0.0229 | 0.9929 | 0.0075 | 0.0013 | 0.0054 |  | |
| childhood maltreatment | Platelet count | 22139419 | | haemotological | Eur | 0.0047 | 0.0542 | 0.086 | 0.9315 | 0.1198 | | | 0.0123 | 0.9898 | 0.0105 | 0.0106 | 0.0055 |  | |
| childhood maltreatment | Systemic lupus erythematosus | 26502338 | | autoimmune | Eur | 0.0062 | 0.0684 | 0.0909 | 0.9276 | 0.41 | | | 0.0702 | 1.0982 | 0.0117 | 0.005 | 0.0059 |  | |
| childhood maltreatment | Concentration of large HDL particles | 27005778 | | metabolites | Eur | -0.0125 | 0.09 | -0.1392 | 0.8893 | 0.1207 | | | 0.0329 | 1.0047 | 0.0175 | -0.0076 | 0.0052 |  | |
| childhood maltreatment | Mean Pallidum | 25607358 | | brain_volume | Eur | 0.015 | 0.0923 | 0.1628 | 0.8707 | 0.1673 | | | 0.0431 | 0.9777 | 0.0074 | -0.0004 | 0.0056 |  | |
| childhood maltreatment | Phospholipids in large HDL | 27005778 | | metabolites | Eur | -0.0164 | 0.0919 | -0.1783 | 0.8585 | 0.1157 | | | 0.0327 | 1.0035 | 0.0163 | -0.0071 | 0.0052 |  | |
| childhood maltreatment | Total Cholesterol | 20686565 | | lipids | Eur | -0.0096 | 0.0485 | -0.1982 | 0.8429 | 0.1323 | | | 0.0255 | 1.0308 | 0.039 | 0.0023 | 0.0061 |  | |
| childhood maltreatment | Child birth length | 25281659 | | anthropometric | Eur | -0.0157 | 0.0756 | -0.2077 | 0.8355 | 0.1745 | | | 0.0235 | 0.991 | 0.0074 | -0.0021 | 0.0055 |  | |
| childhood maltreatment | Free cholesterol in large HDL | 27005778 | | metabolites | Eur | -0.0203 | 0.0941 | -0.2159 | 0.829 | 0.0963 | | | 0.0305 | 1.0144 | 0.0165 | -0.008 | 0.0051 |  | |
| childhood maltreatment | Total lipids in large HDL | 27005778 | | metabolites | Eur | -0.0206 | 0.0902 | -0.2286 | 0.8192 | 0.1176 | | | 0.0334 | 1.0065 | 0.0173 | -0.0073 | 0.0052 |  | |
| childhood maltreatment | Cholesterol esters in large HDL | 27005778 | | metabolites | Eur | -0.0209 | 0.091 | -0.2302 | 0.8179 | 0.1115 | | | 0.0337 | 1.0064 | 0.0166 | -0.007 | 0.005 |  | |
| childhood maltreatment | Birth weight | 27680694 | | anthropometric | Eur | 0.0098 | 0.0412 | 0.2381 | 0.8118 | 0.0994 | | | 0.007 | 1.0556 | 0.012 | 0.0025 | 0.0061 |  | |
| childhood maltreatment | Forced expiratory volume in 1 second (FEV1)/Forced Vital capacity(FVC) | 26635082 | | lung_function | Eur | -0.0175 | 0.0719 | -0.2436 | 0.8075 | 0.1146 | | | 0.016 | 0.9814 | 0.0081 | 0.0004 | 0.005 |  | |
| childhood maltreatment | Total cholesterol in large HDL | 27005778 | | metabolites | Eur | -0.0289 | 0.0945 | -0.3062 | 0.7595 | 0.0936 | | | 0.0309 | 1.0131 | 0.017 | -0.0065 | 0.0051 |  | |
| childhood maltreatment | Forced expiratory volume in 1 second (FEV1)/Forced Vital capacity(FVC) | 21946350 | | lung_function | Eur | -0.0218 | 0.068 | -0.3204 | 0.7487 | 0.1166 | | | 0.0142 | 0.9386 | 0.0089 | -0.0037 | 0.0057 |  | |
| childhood maltreatment | Creatinine | 27005778 | | metabolites | Eur | 0.031 | 0.0952 | 0.3258 | 0.7446 | 0.1041 | | | 0.0258 | 1.0186 | 0.0082 | -0.0082 | 0.0052 |  | |
| childhood maltreatment | Triglycerides in chylomicrons and largest VLDL particles | 27005778 | | metabolites | Eur | 0.0303 | 0.0921 | 0.3287 | 0.7424 | 0.0891 | | | 0.0288 | 0.9926 | 0.0077 | 0.0019 | 0.005 |  | |
| childhood maltreatment | Mean diameter for VLDL particles | 27005778 | | metabolites | Eur | 0.0364 | 0.0832 | 0.4373 | 0.6619 | 0.1354 | | | 0.0368 | 0.9902 | 0.0096 | -0.0016 | 0.0052 |  | |
| childhood maltreatment | Infant head circumference | 22504419 | | anthropometric | Eur | -0.0426 | 0.0963 | -0.4425 | 0.6581 | 0.2333 | | | 0.0476 | 0.9897 | 0.0073 | -0.0053 | 0.0056 |  | |
| childhood maltreatment | Anorexia Nervosa | 24514567 | | psychiatric | Eur | -0.0244 | 0.0519 | -0.4699 | 0.6384 | 0.4768 | | | 0.0324 | 0.9308 | 0.0079 | 0.0107 | 0.0055 |  | |
| childhood maltreatment | Mean Putamen | 25607358 | | brain_volume | Eur | 0.0367 | 0.0745 | 0.4929 | 0.6221 | 0.3151 | | | 0.0477 | 0.948 | 0.0078 | -0.0051 | 0.0057 |  | |
| childhood maltreatment | Peak expiratory flow | 0 | | lung_function | Eur | -0.0457 | 0.0872 | -0.5247 | 0.5998 | 0.0998 | | | 0.0214 | 0.9705 | 0.0075 | -0.0021 | 0.0052 |  | |
| childhood maltreatment | Valine | 27005778 | | metabolites | Eur | -0.056 | 0.1027 | -0.5451 | 0.5857 | 0.0652 | | | 0.0209 | 1.0141 | 0.0086 | -3.9379e-05 | 0.0053 |  | |
| childhood maltreatment | Phospholipids in large VLDL | 27005778 | | metabolites | Eur | 0.0487 | 0.0889 | 0.5481 | 0.5836 | 0.1054 | | | 0.0318 | 0.9941 | 0.0075 | 0.0011 | 0.005 |  | |
| childhood maltreatment | Concentration of chylomicrons and largest VLDL particles | 27005778 | | metabolites | Eur | 0.0529 | 0.0962 | 0.5495 | 0.5827 | 0.1005 | | | 0.0288 | 0.9947 | 0.0078 | 0.0006 | 0.0052 |  | |
| childhood maltreatment | Cholesterol esters in large VLDL | 27005778 | | metabolites | Eur | 0.0449 | 0.0797 | 0.5632 | 0.5733 | 0.1579 | | | 0.0357 | 0.9805 | 0.0078 | -0.0009 | 0.0049 |  | |
| childhood maltreatment | Isoleucine | 27005778 | | metabolites | Eur | -0.0645 | 0.111 | -0.5817 | 0.5608 | 0.0724 | | | 0.0249 | 0.9941 | 0.0075 | 0.002 | 0.0057 |  | |
| childhood maltreatment | Concentration of large VLDL particles | 27005778 | | metabolites | Eur | 0.0513 | 0.0879 | 0.5832 | 0.5598 | 0.1271 | | | 0.0361 | 0.9756 | 0.0078 | -0.0012 | 0.0051 |  | |
| childhood maltreatment | FEV1/FVC | 0 | | lung_function | Eur | 0.0333 | 0.0565 | 0.5891 | 0.5558 | 0.0982 | | | 0.0106 | 0.9922 | 0.0084 | -0.0056 | 0.0059 |  | |
| childhood maltreatment | Peak expiratory flow | 0 | | lung_function | Eur | -0.0173 | 0.0293 | -0.5914 | 0.5543 | 0.1396 | | | 0.009 | 0.9936 | 0.0245 | -0.0088 | 0.0063 |  | |
| childhood maltreatment | Ulcerative colitis | 26192919 | | autoimmune | Eur | 0.03 | 0.0498 | 0.6019 | 0.5472 | 0.2477 | | | 0.0339 | 1.0581 | 0.0114 | -0.0048 | 0.0055 |  | |
| childhood maltreatment | Apolipoprotein A-I | 27005778 | | metabolites | Eur | 0.0766 | 0.1258 | 0.6093 | 0.5423 | 0.0726 | | | 0.0345 | 1.0057 | 0.014 | -0.0058 | 0.0059 |  | |
| childhood maltreatment | Urinary albumin-to-creatinine ratio (non-diabetes) | 26631737 | | kidney | Eur | 0.0548 | 0.0892 | 0.614 | 0.5392 | 0.0534 | | | 0.0109 | 0.995 | 0.0075 | 0.0009 | 0.005 |  | |
| childhood maltreatment | Forced expiratory volume in 1 second (FEV1) | 28166213 | | lung_function | Eur | -0.0302 | 0.047 | -0.6414 | 0.5213 | 0.2624 | | | 0.0192 | 0.9796 | 0.0089 | 0.0001 | 0.0057 |  | |
| childhood maltreatment | Mean diameter for HDL particles | 27005778 | | metabolites | Eur | -0.0612 | 0.0944 | -0.6488 | 0.5164 | 0.1062 | | | 0.033 | 1.0148 | 0.0216 | -0.0031 | 0.0053 |  | |
| childhood maltreatment | Age at Menopause | 26414677 | | reproductive | Eur | -0.0331 | 0.0511 | -0.6489 | 0.5164 | 0.1372 | | | 0.0164 | 0.9936 | 0.0161 | -0.002 | 0.0054 |  | |
| childhood maltreatment | Phospholipids in medium HDL | 27005778 | | metabolites | Eur | 0.0745 | 0.1126 | 0.6617 | 0.5082 | 0.0696 | | | 0.0249 | 0.9944 | 0.0089 | -0.0068 | 0.0055 |  | |
| childhood maltreatment | Forced expiratory volume in 1 second (FEV1)/Forced Vital capacity(FVC) | 28166213 | | lung_function | Eur | 0.032 | 0.0477 | 0.6704 | 0.5026 | 0.2623 | | | 0.0216 | 0.977 | 0.0099 | -0.0011 | 0.0057 |  | |
| childhood maltreatment | Peak expiratory flow | 0 | | lung_function | Eur | -0.0212 | 0.0315 | -0.6722 | 0.5014 | 0.1446 | | | 0.0093 | 0.9934 | 0.0245 | -0.0079 | 0.0071 |  | |
| childhood maltreatment | Triglycerides in large VLDL | 27005778 | | metabolites | Eur | 0.0603 | 0.0884 | 0.6822 | 0.4951 | 0.1051 | | | 0.0298 | 0.9911 | 0.0073 | 0.0003 | 0.005 |  | |
| childhood maltreatment | Ferritin | 25352340 | | metal | Eur | -0.0766 | 0.1073 | -0.7139 | 0.4753 | 0.0936 | | | 0.027 | 1.0276 | 0.0096 | 0.0071 | 0.0068 |  | |
| childhood maltreatment | 18:2 linoleic acid (LA) | 27005778 | | metabolites | Eur | 0.0785 | 0.1099 | 0.7143 | 0.4751 | 0.14 | | | 0.05 | 1.0006 | 0.0104 | 0.0009 | 0.0055 |  | |
| childhood maltreatment | Forced Vital capacity(FVC) | 26635082 | | lung_function | Eur | -0.0443 | 0.0594 | -0.7452 | 0.4561 | 0.148 | | | 0.0168 | 0.9857 | 0.008 | -0.0028 | 0.0053 |  | |
| childhood maltreatment | 22:6 docosahexaenoic acid | 27005778 | | metabolites | Eur | 0.0792 | 0.1028 | 0.77 | 0.4413 | 0.1262 | | | 0.038 | 0.9988 | 0.008 | -0.0047 | 0.0048 |  | |
| childhood maltreatment | HbA1C | 20858683 | | glycemic | Eur | 0.0703 | 0.0878 | 0.7998 | 0.4238 | 0.0624 | | | 0.0124 | 1.0028 | 0.008 | 0.0021 | 0.0054 |  | |
| childhood maltreatment | Autism spectrum disorder | 0 | | psychiatric | Eur | 0.0585 | 0.0695 | 0.8424 | 0.3995 | 0.3898 | | | 0.0567 | 0.9836 | 0.0077 | 0.0079 | 0.0048 |  | |
| childhood maltreatment | Average number of double bonds in a fatty acid chain | 27005778 | | metabolites | Eur | 0.0662 | 0.0768 | 0.8616 | 0.3889 | 0.1847 | | | 0.0558 | 0.9997 | 0.0085 | -0.0029 | 0.0049 |  | |
| childhood maltreatment | Phospholipids in chylomicrons and largest VLDL particles | 27005778 | | metabolites | Eur | 0.082 | 0.0946 | 0.8665 | 0.3862 | 0.0915 | | | 0.0278 | 0.9878 | 0.0077 | 0.0004 | 0.005 |  | |
| childhood maltreatment | Alanine | 27005778 | | metabolites | Eur | 0.0865 | 0.0986 | 0.8772 | 0.3804 | 0.0879 | | | 0.0275 | 1.0106 | 0.0082 | -0.0014 | 0.0054 |  | |
| childhood maltreatment | Parkinsons disease | 19915575 | | neurological | Eur | 0.0608 | 0.0685 | 0.8882 | 0.3744 | 0.4126 | | | 0.1184 | 1.1234 | 0.0089 | -0.0011 | 0.0053 |  | |
| childhood maltreatment | Triglycerides in very large VLDL | 27005778 | | metabolites | Eur | 0.0743 | 0.0836 | 0.8886 | 0.3742 | 0.1131 | | | 0.0307 | 0.9846 | 0.0082 | -0.0004 | 0.0052 |  | |
| childhood maltreatment | Leptin_adjBMI | 26833098 | | hormone | Eur | -0.0744 | 0.0797 | -0.9325 | 0.3511 | 0.0938 | | | 0.0184 | 1.0043 | 0.0077 | 0.009 | 0.0052 |  | |
| childhood maltreatment | Phospholipids in very large HDL | 27005778 | | metabolites | Eur | -0.1078 | 0.1156 | -0.933 | 0.3508 | 0.0776 | | | 0.0322 | 1.0145 | 0.019 | -0.0028 | 0.0052 |  | |
| childhood maltreatment | Total lipids in large VLDL | 27005778 | | metabolites | Eur | 0.0809 | 0.0846 | 0.9569 | 0.3386 | 0.1306 | | | 0.0308 | 0.9881 | 0.0071 | -0.0005 | 0.0049 |  | |
| childhood maltreatment | Forced Vital capacity(FVC) | 28166213 | | lung_function | Eur | -0.046 | 0.0479 | -0.9606 | 0.3368 | 0.2612 | | | 0.0177 | 0.9819 | 0.0086 | -0.0023 | 0.0057 |  | |
| childhood maltreatment | Total lipids in chylomicrons and largest VLDL particles | 27005778 | | metabolites | Eur | 0.0917 | 0.0954 | 0.9613 | 0.3364 | 0.1105 | | | 0.0288 | 0.9946 | 0.0076 | -0.0006 | 0.0049 |  | |
| childhood maltreatment | Cholesterol esters in medium VLDL | 27005778 | | metabolites | Eur | 0.0856 | 0.0853 | 1.003 | 0.3159 | 0.1454 | | | 0.0387 | 0.9905 | 0.0086 | -0.0018 | 0.0051 |  | |
| childhood maltreatment | Inflammatory Bowel Disease (Euro) | 26192919 | | autoimmune | Eur | 0.0437 | 0.0435 | 1.0057 | 0.3145 | 0.3203 | | | 0.0362 | 1.0694 | 0.0126 | -0.0066 | 0.006 |  | |
| childhood maltreatment | Free cholesterol in medium HDL | 27005778 | | metabolites | Eur | 0.1193 | 0.1165 | 1.0239 | 0.3059 | 0.068 | | | 0.0245 | 0.998 | 0.0096 | -0.0093 | 0.0056 |  | |
| childhood maltreatment | Total cholesterol in large VLDL | 27005778 | | metabolites | Eur | 0.0918 | 0.0894 | 1.0275 | 0.3042 | 0.1099 | | | 0.0301 | 0.9955 | 0.0076 | -0.0013 | 0.005 |  | |
| childhood maltreatment | Phospholipids in very large VLDL | 27005778 | | metabolites | Eur | 0.0992 | 0.096 | 1.0335 | 0.3013 | 0.1019 | | | 0.029 | 0.9883 | 0.0077 | -0.001 | 0.0052 |  | |
| childhood maltreatment | Concentration of very large VLDL particles | 27005778 | | metabolites | Eur | 0.093 | 0.0897 | 1.0369 | 0.2998 | 0.1208 | | | 0.0314 | 0.9845 | 0.008 | -0.0005 | 0.0052 |  | |
| childhood maltreatment | FEV1/FVC | 0 | | lung_function | Eur | 0.032 | 0.0305 | 1.0513 | 0.2931 | 0.1681 | | | 0.0116 | 0.9921 | 0.0287 | -0.0081 | 0.007 |  | |
| childhood maltreatment | Serum total triglycerides | 27005778 | | metabolites | Eur | 0.0948 | 0.087 | 1.0888 | 0.2762 | 0.1199 | | | 0.0372 | 0.9958 | 0.0088 | -0.0008 | 0.0053 |  | |
| childhood maltreatment | Phospholipids in medium VLDL | 27005778 | | metabolites | Eur | 0.0999 | 0.0901 | 1.1096 | 0.2672 | 0.1043 | | | 0.0347 | 1.0003 | 0.0081 | -0.0019 | 0.005 |  | |
| childhood maltreatment | Total lipids in medium VLDL | 27005778 | | metabolites | Eur | 0.0917 | 0.0814 | 1.1266 | 0.2599 | 0.1341 | | | 0.0361 | 0.9889 | 0.0077 | -0.0028 | 0.005 |  | |
| childhood maltreatment | Mean platelet volume | 22139419 | | haemotological | Eur | 0.0715 | 0.0627 | 1.1402 | 0.2542 | 0.3235 | | | 0.0535 | 0.9832 | 0.012 | -0.0045 | 0.0057 |  | |
| childhood maltreatment | Urinary albumin-to-creatinine ratio | 26631737 | | kidney | Eur | 0.1019 | 0.0889 | 1.1465 | 0.2516 | 0.0457 | | | 0.009 | 0.9962 | 0.0071 | 0.0022 | 0.0053 |  | |
| childhood maltreatment | Forced expiratory volume in 1 second (FEV1) | 26635082 | | lung_function | Eur | -0.0697 | 0.0601 | -1.1594 | 0.2463 | 0.139 | | | 0.017 | 0.9938 | 0.0077 | -0.0001 | 0.0052 |  | |
| childhood maltreatment | FEV1/FVC | 0 | | lung_function | Eur | 0.0348 | 0.0299 | 1.1622 | 0.2451 | 0.1947 | | | 0.0134 | 0.9632 | 0.0296 | -0.0075 | 0.0071 |  | |
| childhood maltreatment | Concentration of medium VLDL particles | 27005778 | | metabolites | Eur | 0.0965 | 0.0808 | 1.1949 | 0.2321 | 0.1379 | | | 0.0376 | 0.985 | 0.0079 | -0.0032 | 0.005 |  | |
| childhood maltreatment | Free cholesterol in large VLDL | 27005778 | | metabolites | Eur | 0.1069 | 0.0893 | 1.1967 | 0.2314 | 0.1159 | | | 0.0314 | 0.9927 | 0.0078 | -0.0026 | 0.0051 |  | |
| childhood maltreatment | Omega-3 fatty acids | 27005778 | | metabolites | Eur | 0.1324 | 0.1102 | 1.2016 | 0.2295 | 0.1369 | | | 0.0441 | 0.9973 | 0.0087 | -0.0046 | 0.0051 |  | |
| childhood maltreatment | Total lipids in very large VLDL | 27005778 | | metabolites | Eur | 0.1016 | 0.0842 | 1.2073 | 0.2273 | 0.1324 | | | 0.0313 | 0.9809 | 0.0081 | -0.0013 | 0.0049 |  | |
| childhood maltreatment | Alzheimers disease | 24162737 | | neurological | Eur | -0.1186 | 0.0964 | -1.23 | 0.2187 | 0.0485 | | | 0.0255 | 1.0634 | 0.0357 | 0.0074 | 0.0054 |  | |
| childhood maltreatment | Phospholipids in IDL | 27005778 | | metabolites | Eur | 0.1646 | 0.1334 | 1.2333 | 0.2175 | 0.0737 | | | 0.0421 | 1.0178 | 0.0196 | -0.0013 | 0.0059 |  | |
| childhood maltreatment | Total cholesterol in medium VLDL | 27005778 | | metabolites | Eur | 0.112 | 0.0903 | 1.2402 | 0.2149 | 0.1161 | | | 0.037 | 0.9978 | 0.0087 | -0.0034 | 0.0052 |  | |
| childhood maltreatment | Mean Hippocampus | 25607358 | | brain_volume | Eur | -0.1294 | 0.1011 | -1.2795 | 0.2007 | 0.162 | | | 0.0417 | 0.9818 | 0.0078 | -0.0033 | 0.0059 |  | |
| childhood maltreatment | Neo-openness to experience | 21173776 | | personality | Eur | 0.1307 | 0.1013 | 1.2906 | 0.1968 | 0.1099 | | | 0.0268 | 0.9901 | 0.0069 | 0.0007 | 0.0051 |  | |
| childhood maltreatment | Leptin_not_adjBMI | 26833098 | | hormone | Eur | 0.1026 | 0.0794 | 1.2916 | 0.1965 | 0.0968 | | | 0.0165 | 0.9963 | 0.0074 | 0.0101 | 0.0054 |  | |
| childhood maltreatment | Height_2010 | 20881960 | | anthropometric | Eur | 0.0437 | 0.0336 | 1.3007 | 0.1934 | 0.2862 | | | 0.0181 | 1.0289 | 0.0224 | -0.0098 | 0.0064 |  | |
| childhood maltreatment | Child birth weight | 23202124 | | anthropometric | Eur | 0.1028 | 0.0789 | 1.3021 | 0.1929 | 0.12 | | | 0.019 | 1 | 0.0066 | -0.0049 | 0.0047 |  | |
| childhood maltreatment | Triglycerides in small VLDL | 27005778 | | metabolites | Eur | 0.1183 | 0.0902 | 1.3124 | 0.1894 | 0.1166 | | | 0.0373 | 0.9975 | 0.0087 | -0.0008 | 0.0054 |  | |
| childhood maltreatment | Triglycerides in IDL | 27005778 | | metabolites | Eur | 0.1326 | 0.101 | 1.3131 | 0.1892 | 0.1111 | | | 0.0359 | 1.0108 | 0.0166 | 0.0009 | 0.0055 |  | |
| childhood maltreatment | Crohns disease | 26192919 | | autoimmune | Eur | 0.065 | 0.0494 | 1.3167 | 0.1879 | 0.4911 | | | 0.0636 | 1.033 | 0.015 | -0.008 | 0.0058 |  | |
| childhood maltreatment | Primary biliary cirrhosis | 26394269 | | autoimmune | Eur | 0.1037 | 0.0787 | 1.3174 | 0.1877 | 0.3718 | | | 0.0638 | 1.0073 | 0.0115 | 0.0012 | 0.0062 |  | |
| childhood maltreatment | ICV | 25607358 | | brain_volume | Eur | -0.1331 | 0.1006 | -1.3228 | 0.1859 | 0.1834 | | | 0.0463 | 1.0014 | 0.0074 | 0.0005 | 0.0054 |  | |
| childhood maltreatment | Triglycerides in medium VLDL | 27005778 | | metabolites | Eur | 0.125 | 0.093 | 1.3451 | 0.1786 | 0.0926 | | | 0.0317 | 1.0006 | 0.0076 | -0.0023 | 0.005 |  | |
| childhood maltreatment | Free cholesterol in large LDL | 27005778 | | metabolites | Eur | 0.1652 | 0.1225 | 1.349 | 0.1773 | 0.0794 | | | 0.0487 | 1.0136 | 0.024 | -0.0034 | 0.0057 |  | |
| childhood maltreatment | Sitting height ratio | 25865494 | | anthropometric | Eur | -0.0928 | 0.0681 | -1.3619 | 0.1732 | 0.2222 | | | 0.0285 | 0.9804 | 0.0087 | 0.0066 | 0.0057 |  | |
| childhood maltreatment | Free cholesterol in IDL | 27005778 | | metabolites | Eur | 0.1673 | 0.1218 | 1.3734 | 0.1696 | 0.0882 | | | 0.0424 | 1.0151 | 0.0203 | -0.0017 | 0.006 |  | |
| childhood maltreatment | Free cholesterol in medium VLDL | 27005778 | | metabolites | Eur | 0.1302 | 0.0943 | 1.3801 | 0.1675 | 0.1031 | | | 0.035 | 0.9999 | 0.0083 | -0.0037 | 0.0052 |  | |
| childhood maltreatment | Sleep duration | 27494321 | | sleeping | Eur | -0.0868 | 0.0625 | -1.3892 | 0.1648 | 0.0554 | | | 0.0053 | 1.0237 | 0.0085 | -0.0076 | 0.0058 |  | |
| childhood maltreatment | Phospholipids in very small VLDL | 27005778 | | metabolites | Eur | 0.1656 | 0.1181 | 1.4024 | 0.1608 | 0.0958 | | | 0.0396 | 1.0129 | 0.0163 | -0.0021 | 0.0058 |  | |
| childhood maltreatment | Total cholesterol in small VLDL | 27005778 | | metabolites | Eur | 0.1597 | 0.1113 | 1.4349 | 0.1513 | 0.0903 | | | 0.0331 | 1.0088 | 0.0103 | -0.0033 | 0.0056 |  | |
| childhood maltreatment | Phospholipids in large LDL | 27005778 | | metabolites | Eur | 0.1869 | 0.1291 | 1.4478 | 0.1477 | 0.0767 | | | 0.0449 | 1.0143 | 0.0216 | -0.0036 | 0.0057 |  | |
| childhood maltreatment | Chronotype | 27494321 | | sleeping | Eur | -0.0769 | 0.0529 | -1.452 | 0.1465 | 0.101 | | | 0.0062 | 1.0222 | 0.0089 | 0.0025 | 0.0061 |  | |
| childhood maltreatment | Total cholesterol in LDL | 27005778 | | metabolites | Eur | 0.1794 | 0.1231 | 1.4576 | 0.145 | 0.0841 | | | 0.0512 | 1.0106 | 0.0243 | -0.0042 | 0.0059 |  | |
| childhood maltreatment | Celiac disease | 20190752 | | autoimmune | Eur | -0.122 | 0.0818 | -1.4912 | 0.1359 | 0.3099 | | | 0.0494 | 1.0651 | 0.011 | 0.0084 | 0.0075 |  | |
| childhood maltreatment | Mean Caudate | 25607358 | | brain_volume | Eur | 0.1301 | 0.0866 | 1.5021 | 0.1331 | 0.2613 | | | 0.0409 | 0.9687 | 0.0071 | -0.0009 | 0.0061 |  | |
| childhood maltreatment | Concentration of IDL particles | 27005778 | | metabolites | Eur | 0.1899 | 0.1263 | 1.5041 | 0.1325 | 0.0889 | | | 0.0439 | 1.0156 | 0.0186 | -0.002 | 0.0059 |  | |
| childhood maltreatment | Total lipids in IDL | 27005778 | | metabolites | Eur | 0.1924 | 0.1275 | 1.5096 | 0.1311 | 0.0894 | | | 0.0452 | 1.016 | 0.0193 | -0.0025 | 0.0059 |  | |
| childhood maltreatment | Concentration of small VLDL particles | 27005778 | | metabolites | Eur | 0.1307 | 0.086 | 1.5194 | 0.1286 | 0.1466 | | | 0.0388 | 0.9938 | 0.0088 | -0.0027 | 0.0054 |  | |
| childhood maltreatment | Free cholesterol in small VLDL | 27005778 | | metabolites | Eur | 0.1553 | 0.0997 | 1.557 | 0.1195 | 0.1102 | | | 0.035 | 1.0036 | 0.0097 | -0.0034 | 0.0058 |  | |
| childhood maltreatment | Total cholesterol in large LDL | 27005778 | | metabolites | Eur | 0.197 | 0.1252 | 1.5731 | 0.1157 | 0.0807 | | | 0.047 | 1.0116 | 0.0229 | -0.0047 | 0.0057 |  | |
| childhood maltreatment | Age of smoking initiation | 20418890 | | smoking_behaviour | Eur | -0.1874 | 0.1181 | -1.5862 | 0.1127 | 0.0539 | | | 0.0191 | 1.0033 | 0.0071 | -0.0053 | 0.0049 |  | |
| childhood maltreatment | Total lipids in small VLDL | 27005778 | | metabolites | Eur | 0.1458 | 0.0917 | 1.5892 | 0.112 | 0.1406 | | | 0.0388 | 0.9958 | 0.0091 | -0.0032 | 0.0056 |  | |
| childhood maltreatment | Concentration of large LDL particles | 27005778 | | metabolites | Eur | 0.2044 | 0.1278 | 1.6 | 0.1096 | 0.0915 | | | 0.0511 | 1.0129 | 0.0218 | -0.0039 | 0.006 |  | |
| childhood maltreatment | Forced expiratory volume in 1 second | 0 | | lung_function | Eur | -0.0804 | 0.0502 | -1.6006 | 0.1095 | 0.1306 | | | 0.0106 | 0.9957 | 0.0096 | -0.0028 | 0.0054 |  | |
| childhood maltreatment | Total cholesterol in IDL | 27005778 | | metabolites | Eur | 0.208 | 0.129 | 1.6125 | 0.1069 | 0.0901 | | | 0.0465 | 1.0148 | 0.0201 | -0.0042 | 0.0058 |  | |
| childhood maltreatment | Total lipids in very small VLDL | 27005778 | | metabolites | Eur | 0.174 | 0.1074 | 1.6203 | 0.1052 | 0.1094 | | | 0.0372 | 1.0113 | 0.014 | -0.0011 | 0.0055 |  | |
| childhood maltreatment | Total lipids in large LDL | 27005778 | | metabolites | Eur | 0.208 | 0.1279 | 1.6271 | 0.1037 | 0.0923 | | | 0.0522 | 1.0112 | 0.0224 | -0.0046 | 0.006 |  | |
| childhood maltreatment | Total cholesterol in medium LDL | 27005778 | | metabolites | Eur | 0.1977 | 0.1211 | 1.6328 | 0.1025 | 0.0824 | | | 0.047 | 1.0107 | 0.0223 | -0.0053 | 0.0058 |  | |
| childhood maltreatment | Apolipoprotein B | 27005778 | | metabolites | Eur | 0.1979 | 0.1207 | 1.6392 | 0.1012 | 0.0878 | | | 0.0409 | 1.0096 | 0.0151 | -0.0041 | 0.0055 |  | |
| childhood maltreatment | Total cholesterol in small LDL | 27005778 | | metabolites | Eur | 0.208 | 0.1263 | 1.6466 | 0.0996 | 0.0814 | | | 0.0447 | 1.0146 | 0.0195 | -0.0042 | 0.0059 |  | |
| childhood maltreatment | Triglycerides in very small VLDL | 27005778 | | metabolites | Eur | 0.1487 | 0.0898 | 1.6568 | 0.0976 | 0.1369 | | | 0.0377 | 1.0009 | 0.0108 | -0.0013 | 0.0053 |  | |
| childhood maltreatment | Extreme waist-to-hip ratio | 23563607 | | anthropometric | Eur | 0.141 | 0.0848 | 1.6627 | 0.0964 | 0.3611 | | | 0.0576 | 0.9771 | 0.0085 | -0.0033 | 0.0068 |  | |
| childhood maltreatment | Phospholipids in small VLDL | 27005778 | | metabolites | Eur | 0.1714 | 0.1017 | 1.685 | 0.092 | 0.1111 | | | 0.0365 | 1.0035 | 0.0095 | -0.0036 | 0.0056 |  | |
| childhood maltreatment | Concentration of very small VLDL particles | 27005778 | | metabolites | Eur | 0.1677 | 0.099 | 1.6946 | 0.0901 | 0.1194 | | | 0.0373 | 1.0061 | 0.0135 | -0.0014 | 0.0053 |  | |
| childhood maltreatment | Concentration of medium LDL particles | 27005778 | | metabolites | Eur | 0.2116 | 0.1241 | 1.7047 | 0.0882 | 0.094 | | | 0.0495 | 1.0093 | 0.0204 | -0.0045 | 0.006 |  | |
| childhood maltreatment | Total lipids in medium LDL | 27005778 | | metabolites | Eur | 0.2107 | 0.1232 | 1.7098 | 0.0873 | 0.0958 | | | 0.0509 | 1.0075 | 0.021 | -0.0048 | 0.006 |  | |
| childhood maltreatment | Cholesterol esters in medium LDL | 27005778 | | metabolites | Eur | 0.2062 | 0.1206 | 1.7103 | 0.0872 | 0.0964 | | | 0.0503 | 1.0072 | 0.021 | -0.0053 | 0.006 |  | |
| childhood maltreatment | Cholesterol esters in large LDL | 27005778 | | metabolites | Eur | 0.2163 | 0.1258 | 1.7196 | 0.0855 | 0.0946 | | | 0.0516 | 1.0088 | 0.022 | -0.0058 | 0.0059 |  | |
| childhood maltreatment | Phospholipids in medium LDL | 27005778 | | metabolites | Eur | 0.2142 | 0.1219 | 1.7569 | 0.0789 | 0.0861 | | | 0.0429 | 1.006 | 0.0195 | -0.0062 | 0.0058 |  | |
| childhood maltreatment | Forced expiratory volume in 1 second (FEV1) | 21946350 | | lung_function | Eur | -0.1721 | 0.0975 | -1.7646 | 0.0776 | 0.1712 | | | 0.0233 | 0.8824 | 0.0136 | -0.0036 | 0.0103 |  | |
| childhood maltreatment | Total lipids in small LDL | 27005778 | | metabolites | Eur | 0.2127 | 0.1199 | 1.7741 | 0.0761 | 0.097 | | | 0.0465 | 1.0071 | 0.0179 | -0.0041 | 0.006 |  | |
| childhood maltreatment | Forced expiratory volume in 1 second | 0 | | lung_function | Eur | -0.0544 | 0.0306 | -1.7773 | 0.0755 | 0.1787 | | | 0.0075 | 0.9774 | 0.0178 | -0.0146 | 0.0075 |  | |
| childhood maltreatment | Concentration of small LDL particles | 27005778 | | metabolites | Eur | 0.2119 | 0.1174 | 1.8053 | 0.071 | 0.1031 | | | 0.0445 | 1.0065 | 0.0162 | -0.0045 | 0.006 |  | |
| childhood maltreatment | Forced expiratory volume in 1 second | 0 | | lung_function | Eur | -0.0538 | 0.0293 | -1.8326 | 0.0669 | 0.1673 | | | 0.0068 | 1.012 | 0.0192 | -0.0155 | 0.007 |  | |
| childhood maltreatment | Extreme height | 23563607 | | anthropometric | Eur | 0.0854 | 0.0432 | 1.9779 | 0.0479 | 1.2301 | | | 0.1061 | 1.0365 | 0.0176 | -0.0099 | 0.007 |  | |
| childhood maltreatment | Fasting glucose main effect | 22581228 | | glycemic | Eur | 0.1288 | 0.0624 | 2.0647 | 0.039 | 0.0999 | | | 0.0199 | 0.996 | 0.0113 | -0.005 | 0.0051 |  | |
| childhood maltreatment | Asthma | 17611496 | | autoimmune | Eur | 0.1568 | 0.0743 | 2.1114 | 0.0347 | 0.1353 | | | 0.0275 | 1.0029 | 0.0088 | 0.0019 | 0.0056 |  | |
| childhood maltreatment | Forced vital capacity | 0 | | lung_function | Eur | -0.1081 | 0.0498 | -2.1685 | 0.0301 | 0.1173 | | | 0.0101 | 1.0033 | 0.01 | -0.0019 | 0.0053 |  | |
| childhood maltreatment | HOMA-B | 20081858 | | glycemic | Eur | 0.1682 | 0.0764 | 2.2027 | 0.0276 | 0.0862 | | | 0.0135 | 0.9922 | 0.0068 | 0.0002 | 0.0055 |  |  |
| childhood maltreatment | Urate | 23263486 | | other | Eur | 0.1117 | 0.0496 | 2.2516 | 0.0243 | 0.181 | | | 0.0615 | 0.9475 | 0.0572 | -0.0027 | 0.0053 |  |  |
| childhood maltreatment | Triglycerides | 20686565 | | lipids | Eur | 0.1088 | 0.0462 | 2.3536 | 0.0186 | 0.1731 | | | 0.0311 | 0.9655 | 0.0192 | 0.0014 | 0.0061 |  |  |
| childhood maltreatment | Forced vital capacity | 0 | | lung_function | Eur | -0.0757 | 0.0314 | -2.4099 | 0.016 | 0.1762 | | | 0.0068 | 0.9813 | 0.0176 | -0.0112 | 0.0073 |  |  |
| childhood maltreatment | Childhood IQ | 23358156 | | education | Eur | -0.2278 | 0.0942 | -2.4174 | 0.0156 | 0.2725 | | | 0.0475 | 1.0042 | 0.0101 | -0.0028 | 0.0072 |  |  |
| childhood maltreatment | Forced vital capacity | 0 | | lung_function | Eur | -0.0724 | 0.0296 | -2.4444 | 0.0145 | 0.1632 | | | 0.0062 | 1.0148 | 0.0193 | -0.0133 | 0.007 |  |  |
| childhood maltreatment | Type 2 Diabetes | 22885922 | | glycemic | Eur | 0.1631 | 0.0608 | 2.6802 | 0.0074 | 0.0886 | | | 0.0097 | 1.0098 | 0.0082 | -0.0005 | 0.0057 |  |  |
| childhood maltreatment | Rheumatoid Arthritis | 24390342 | | autoimmune | Eur | 0.1407 | 0.0516 | 2.7285 | 0.0064 | 0.1632 | | | 0.0316 | 1.023 | 0.017 | 0.0056 | 0.0063 |  |  |
| childhood maltreatment | Fasting insulin main effect | 22581228 | | glycemic | Eur | 0.2097 | 0.0731 | 2.8693 | 0.0041 | 0.0673 | | | 0.0106 | 1.0189 | 0.0078 | -0.003 | 0.0052 |  |  |
| childhood maltreatment | Cigarettes smoked per day | 20418890 | | smoking_behaviour | Eur | 0.2756 | 0.0958 | 2.8785 | 0.004 | 0.0637 | | | 0.0164 | 1.0032 | 0.0069 | 0.0066 | 0.0052 |  |  |
| childhood maltreatment | Citrate | 27005778 | | metabolites | Eur | -0.3073 | 0.1037 | -2.9624 | 0.0031 | 0.0716 | | | 0.0214 | 1.0099 | 0.0085 | 0.0024 | 0.005 |  |  |
| childhood maltreatment | Childhood obesity | 22484627 | | anthropometric | Eur | 0.2094 | 0.0661 | 3.1689 | 0.0015 | 0.3969 | | | 0.0474 | 0.9345 | 0.0082 | -0.0008 | 0.0056 |  |  |
| childhood maltreatment | Intelligence | 28530673 | | cognitive | Eur | -0.1412 | 0.0438 | -3.2275 | 0.0012 | 0.1914 | | | 0.011 | 1.0146 | 0.0092 | -0.0228 | 0.0064 |  |  |
| childhood maltreatment | Former vs Current smoker | 20418890 | | smoking_behaviour | Eur | -0.2838 | 0.0868 | -3.2706 | 0.0011 | 0.0612 | | | 0.011 | 1.0013 | 0.007 | -0.0064 | 0.0046 |  |  |
| childhood maltreatment | HOMA-IR | 20081858 | | glycemic | Eur | 0.2782 | 0.0851 | 3.2679 | 0.0011 | 0.0652 | | | 0.0132 | 1.0069 | 0.007 | -0.0026 | 0.005 |  |  |
| childhood maltreatment | Excessive daytime sleepiness | 27992416 | | sleeping | Eur | 0.1819 | 0.0547 | 3.3227 | 0.0009 | 0.0549 | | | 0.0054 | 1.0049 | 0.0077 | 0.0119 | 0.0057 |  |  |
| childhood maltreatment | Squamous cell lung cancer | 27488534 | | cancer | Eur | 0.4301 | 0.1237 | 3.4778 | 0.0005 | 0.0384 | | | 0.0128 | 1.0141 | 0.0079 | -0.0037 | 0.0053 |  |  |
| childhood maltreatment | Neuroticism | 24828478 | | personality | Eur | 0.4032 | 0.113 | 3.5668 | 0.0004 | 0.0128 | | | 0.0034 | 1.017 | 0.0067 | 0.0031 | 0.0052 |  |  |
| childhood maltreatment | Years of schooling 2013 | 23722424 | | education | Eur | -0.1812 | 0.0508 | -3.5705 | 0.0004 | 0.0854 | | | 0.0066 | 1.0202 | 0.0099 | -0.0102 | 0.006 |  |  |
| childhood maltreatment | Parents age at death | 27015805 | | aging | Eur | -0.3318 | 0.0925 | -3.5859 | 0.0003 | 0.0305 | | | 0.0074 | 1.0161 | 0.0071 | -0.0045 | 0.0057 |  |  |
| childhood maltreatment | Bipolar disorder | 21926972 | | psychiatric | Eur | 0.2113 | 0.0589 | 3.5893 | 0.0003 | 0.4393 | | | 0.0387 | 1.0239 | 0.0083 | -0.0068 | 0.0058 |  |  |
| childhood maltreatment | Adiponectin | 22479202 | | cardiometabolic | Mix |  |  |  |  |  | | |  |  |  |  |  | Caution: using this data may yield less robust results due to minor departure of the LD structure |  |
| childhood maltreatment | Body fat | 26833246 | | anthropometric | Mix |  |  |  |  |  | | |  |  |  |  |  | Caution: using this data may yield less robust results due to minor departure of the LD structure |  |
| childhood maltreatment | Coronary artery disease | 26343387 | | cardiometabolic | Mix |  |  |  |  |  | | |  |  |  |  |  | Caution: using this data may yield less robust results due to minor departure of the LD structure |  |
| childhood maltreatment | Eczema | 26482879 | | autoimmune | Mix |  |  |  |  |  | | |  |  |  |  |  | Caution: using this data may yield less robust results due to minor departure of the LD structure |  |
| childhood maltreatment | Mean Accumbens | 25607358 | | brain_volume | Eur |  |  |  |  |  | | |  |  |  |  |  | Caution: using this data may yield results outside bounds due to relative low Z score of the SNP heritability of the trait |  |
| childhood maltreatment | Forearm Bone mineral density | 26367794 | | bone | Mix |  |  |  |  |  | | |  |  |  |  |  | Caution: using this data may yield results outside bounds due to relative low Z score of the SNP heritability of the trait |  |
| childhood maltreatment | Fasting proinsulin | 20081858 | | glycemic | Eur |  |  |  |  |  | | |  |  |  |  |  | Caution: using this data may yield results outside bounds due to relative low Z score of the SNP heritability of the trait |  |
| childhood maltreatment | Femoral Neck bone mineral density | 26367794 | | bone | Mix |  |  |  |  |  | | |  |  |  |  |  | Caution: using this data may yield less robust results due to minor departure of the LD structure |  |
| childhood maltreatment | Chronic Kidney Disease | 26831199 | | kidney | Mix |  |  |  |  |  | | |  |  |  |  |  | Caution: using this data may yield less robust results due to minor departure of the LD structure |  |
| childhood maltreatment | Serum creatinine (non-diabetes) | 26831199 | | kidney | Mix |  |  |  |  |  | | |  |  |  |  |  | Caution: using this data may yield less robust results due to minor departure of the LD structure |  |
| childhood maltreatment | Serum creatinine | 26831199 | | kidney | Mix |  |  |  |  |  | | |  |  |  |  |  | Caution: using this data may yield less robust results due to minor departure of the LD structure |  |
| childhood maltreatment | Serum cystatin c | 26831199 | | kidney | Mix |  |  |  |  |  | | |  |  |  |  |  | Caution: using this data may yield less robust results due to minor departure of the LD structure |  |
| childhood maltreatment | Neo-conscientiousness | 21173776 | | personality | Eur |  |  |  |  |  | | |  |  |  |  |  | Caution: using this data may yield results outside bounds due to relative low Z score of the SNP heritability of the trait |  |
| childhood maltreatment | Lumbar Spine bone mineral density | 26367794 | | bone | Mix |  |  |  |  |  | | |  |  |  |  |  | Caution: using this data may yield less robust results due to minor departure of the LD structure |  |
| childhood maltreatment | 2hr glucose adjusted for BMI | 20081857 | | glycemic | Eur |  |  |  |  |  | | |  |  |  |  |  | Caution: using this data may yield results outside bounds due to relative low Z score of the SNP heritability of the trait |  |
| childhood maltreatment | Heart rate | 23583979 | | haemotological | Mix |  |  |  |  |  | | |  |  |  |  |  | Caution: using this data may yield less robust results due to minor departure of the LD structure |  |
| childhood maltreatment | Serumurate overweight | 25811787 | | uric_acid | Eur |  |  |  |  |  | | |  |  |  |  |  | Caution: using this data may yield results outside bounds due to relative low Z score of the SNP heritability of the trait |  |
| childhood maltreatment | Attention deficit hyperactivity disorder | 20732625 | | psychiatric | Eur |  |  |  |  |  | | |  |  |  |  |  | Caution: using this data may yield results outside bounds due to relative low Z score of the SNP heritability of the trait |  |
| childhood maltreatment | Schizophrenia | 25056061 | | psychiatric | Mix |  |  |  |  |  | | |  |  |  |  |  | Caution: using this data may yield less robust results due to minor departure of the LD structure |  |
| childhood maltreatment | Acetoacetate | 27005778 | | metabolites | Eur |  |  |  |  |  | | |  |  |  |  |  | Caution: using this data may yield results outside bounds due to relative low Z score of the SNP heritability of the trait |  |
| childhood maltreatment | Acetate | 27005778 | | metabolites | Eur |  |  |  |  |  | | |  |  |  |  |  | Caution: using this data may yield results outside bounds due to relative low Z score of the SNP heritability of the trait |  |
| childhood maltreatment | Albumin | 27005778 | | metabolites | Eur |  |  |  |  |  | | |  |  |  |  |  | Caution: using this data may yield results outside bounds due to relative low Z score of the SNP heritability of the trait |  |
| childhood maltreatment | Ratio of bisallylic groups to double bonds | 27005778 | | metabolites | Eur |  |  |  |  |  | | |  |  |  |  |  | Caution: using this data may yield results outside bounds due to relative low Z score of the SNP heritability of the trait |  |
| childhood maltreatment | Ratio of bisallylic groups to total fatty acids | 27005778 | | metabolites | Eur |  |  |  |  |  | | |  |  |  |  |  | Caution: using this data may yield results outside bounds due to relative low Z score of the SNP heritability of the trait |  |
| childhood maltreatment | Average number of methylene groups per a double bond | 27005778 | | metabolites | Eur |  |  |  |  |  | | |  |  |  |  |  | Caution: using this data may yield results outside bounds due to relative low Z score of the SNP heritability of the trait |  |
| childhood maltreatment | Free cholesterol to esterified cholesterol ratio | 27005778 | | metabolites | Eur |  |  |  |  |  | | |  |  |  |  |  | Caution: using this data may yield results outside bounds due to relative low Z score of the SNP heritability of the trait |  |
| childhood maltreatment | Description of average fatty acid chain length |  | |  |  |  |  |  |  |  | | |  |  |  |  |  |  |  |
| childhood maltreatment | Omega-9 and saturated fatty acids | 27005778 | | metabolites | Eur |  |  |  |  |  | | |  |  |  |  |  | Caution: using this data may yield results outside bounds due to relative low Z score of the SNP heritability of the trait |  |
| childhood maltreatment | Free cholesterol | 27005778 | | metabolites | Eur |  |  |  |  |  | | |  |  |  |  |  | Caution: using this data may yield results outside bounds due to relative low Z score of the SNP heritability of the trait |  |
| childhood maltreatment | Glycoprotein acetyls |  | |  |  |  |  |  |  |  | | |  |  |  |  |  |  |  |
| childhood maltreatment | Mean diameter for LDL particles | 27005778 | | metabolites | Eur |  |  |  |  |  | | |  |  |  |  |  | Caution: using this data may yield results outside bounds due to relative low Z score of the SNP heritability of the trait |  |
| childhood maltreatment | Leucine | 27005778 | | metabolites | Eur |  |  |  |  |  | | |  |  |  |  |  | Caution: using this data may yield results outside bounds due to relative low Z score of the SNP heritability of the trait |  |
| childhood maltreatment | Total cholesterol in medium HDL | 27005778 | | metabolites | Eur |  |  |  |  |  | | |  |  |  |  |  | Caution: using this data may yield results outside bounds due to relative low Z score of the SNP heritability of the trait |  |
| childhood maltreatment | Cholesterol esters in medium HDL | 27005778 | | metabolites | Eur |  |  |  |  |  | | |  |  |  |  |  | Caution: using this data may yield results outside bounds due to relative low Z score of the SNP heritability of the trait |  |
| childhood maltreatment | Total lipids in medium HDL | 27005778 | | metabolites | Eur |  |  |  |  |  | | |  |  |  |  |  | Caution: using this data may yield results outside bounds due to relative low Z score of the SNP heritability of the trait |  |
| childhood maltreatment | Concentration of medium HDL particles | 27005778 | | metabolites | Eur |  |  |  |  |  | | |  |  |  |  |  | Caution: using this data may yield results outside bounds due to relative low Z score of the SNP heritability of the trait |  |
| childhood maltreatment | Mono-unsaturated fatty acids | 27005778 | | metabolites | Eur |  |  |  |  |  | | |  |  |  |  |  | Caution: using this data may yield results outside bounds due to relative low Z score of the SNP heritability of the trait |  |
| childhood maltreatment | Phenylalanine | 27005778 | | metabolites | Eur |  |  |  |  |  | | |  |  |  |  |  | Caution: using this data may yield results outside bounds due to relative low Z score of the SNP heritability of the trait |  |
| childhood maltreatment | Total lipids in small HDL | 27005778 | | metabolites | Eur |  |  |  |  |  | | |  |  |  |  |  | Caution: using this data may yield results outside bounds due to relative low Z score of the SNP heritability of the trait |  |
| childhood maltreatment | Triglycerides in small HDL | 27005778 | | metabolites | Eur |  |  |  |  |  | | |  |  |  |  |  | Caution: using this data may yield results outside bounds due to relative low Z score of the SNP heritability of the trait |  |
| childhood maltreatment | Serum total cholesterol | 27005778 | | metabolites | Eur |  |  |  |  |  | | |  |  |  |  |  | Caution: using this data may yield results outside bounds due to relative low Z score of the SNP heritability of the trait |  |
| childhood maltreatment | Tyrosine | 27005778 | | metabolites | Eur |  |  |  |  |  | | |  |  |  |  |  | Caution: using this data may yield results outside bounds due to relative low Z score of the SNP heritability of the trait |  |
| childhood maltreatment | Total cholesterol in very large HDL | 27005778 | | metabolites | Eur |  |  |  |  |  | | |  |  |  |  |  | Caution: using this data may yield results outside bounds due to relative low Z score of the SNP heritability of the trait |  |
| childhood maltreatment | Free cholesterol in very large HDL | 27005778 | | metabolites | Eur |  |  |  |  |  | | |  |  |  |  |  | Caution: using this data may yield results outside bounds due to relative low Z score of the SNP heritability of the trait |  |
| childhood maltreatment | Total lipids in very large HDL | 27005778 | | metabolites | Eur |  |  |  |  |  | | |  |  |  |  |  | Caution: using this data may yield results outside bounds due to relative low Z score of the SNP heritability of the trait |  |
| childhood maltreatment | Concentration of very large HDL particles | 27005778 | | metabolites | Eur |  |  |  |  |  | | |  |  |  |  |  | Caution: using this data may yield results outside bounds due to relative low Z score of the SNP heritability of the trait |  |
| childhood maltreatment | Triglycerides in very large HDL | 27005778 | | metabolites | Eur |  |  |  |  |  | | |  |  |  |  |  | Caution: using this data may yield results outside bounds due to relative low Z score of the SNP heritability of the trait |  |
| childhood maltreatment | Lung cancer (squamous cell) | 24880342 | | cancer | Eur |  |  |  |  |  | | |  |  |  |  |  | Caution: using this data may yield results outside bounds due to relative low Z score of the SNP heritability of the trait |  |
| childhood maltreatment | Multiple sclerosis | 21833088 | | autoimmune | Eur |  |  |  |  |  | | |  |  |  |  |  | Caution: using this data may yield results outside bounds due to relative low Z score of the SNP heritability of the trait |  |
| childhood maltreatment | Transferrin | 25352340 | | metal | Eur |  |  |  |  |  | | |  |  |  |  |  | Caution: using this data may yield results outside bounds due to relative low Z score of the SNP heritability of the trait |  |
| childhood maltreatment | Amyotrophic lateral sclerosis | 27455348 | | neurological | Eur |  |  |  |  |  | | |  |  |  |  |  | Caution: using this data may yield results outside bounds due to relative low Z score of the SNP heritability of the trait |  |
| childhood maltreatment | Lung adenocarcinoma | 27488534 | | cancer | Eur |  |  |  |  |  | | |  |  |  |  |  | Caution: using this data may yield results outside bounds due to relative low Z score of the SNP heritability of the trait |  |
| childhood maltreatment | Difference in height between adolescence and adulthood | | |  |  |  |  |  |  |  | | |  |  |  |  |  |  |  |
| childhood maltreatment | Difference in height between childhood and adulthood | | |  |  |  |  |  |  |  | | |  |  |  |  |  |  |  |
| childhood maltreatment | Height | |  |  |  |  |  |  |  |  | | |  |  |  |  |  |  |  |
| childhood maltreatment | Lumbar spine bone mineral density | | 22504420 | bone | Mix |  |  |  |  |  | | |  |  |  |  |  | Caution: using these data may yield less robust results due to minor departure of the LD structure |  |
| childhood maltreatment | Femoral neck bone mineral density | | 22504420 | bone | Mix |  |  |  |  |  | | |  |  |  |  |  | Caution: using these data may yield less robust results due to minor departure of the LD structure |  |
| childhood maltreatment | Attention deficit hyperactivity disorder (GC) | | 27663945 | psychiatric | Eur |  |  |  |  |  | | |  |  |  |  |  | Caution: using this data may yield results outside bounds due to relative low Z score of the SNP heritability of the trait |  |
| childhood maltreatment | Attention deficit hyperactivity disorder (No GC) | | 27663945 | psychiatric | Eur |  |  |  |  |  | | |  |  |  |  |  | Caution: using this data may yield results outside bounds due to relative low Z score of the SNP heritability of the trait |  |
| childhood maltreatment | Primary sclerosing cholangitis | | 27992413 | autoimmune | Mix |  |  |  |  |  | | |  |  |  |  |  | Caution: using this data may yield results outside bounds due to relative low Z score of the SNP heritability of the trait |  |
| childhood maltreatment | Ischemic stroke | | 26935894 | cardiometabolic | Mix |  |  |  |  |  | | |  |  |  |  |  | Caution: using these data may yield less robust results due to minor departure of the LD structure |  |
