# Supplementary material for: Genomic influences on self-reported childhood maltreatment
Source: Transl Psychiatry. 2020 Jan 27;10:38. doi: 10.1038/s41398-020-0706-0 (PMC7026037; doi:10.1038/s41398-020-0706-0)
Supplement: Supplementary file 4 — Supplementary Table 4 [file 41398_2020_706_MOESM4_ESM.docx]

| **Supplementary Table 4: Top hits from meta-analysis conditioning on the effects of MDD** | | | | | | | | |  |  |  |  |  |
| --- | --- | --- | --- | --- | --- | --- | --- | --- | --- | --- | --- | --- | --- |
|  |  |  |  |  |  |  |  |  |  |  |  |  |  |
| **SNP** | **Proxy SNP¹** | **ld** | **A1** | **A2** | **A1 freq** | **N** | **Beta²** | **SE** | **p** | **Beta_cojo** | **SE_cojo** | **p_cojo** |  |
| rs142346759 | rs13078958 | 0.758 | G | A | 0.069 | 135 569 | 0.030 | 0.006 | 2.12E-06 | 0.031 | 0.006 | 1.41E-06 |  |
| rs1859100, rs10262462 | rs6980093 | 0.992, 0.988 | G | A | 0.411 | 138 601 | -0.017 | 0.003 | 5.36E-08 | -0.018 | 0.003 | 4.61E-08 |  |
| rs917577 | rs12297010 | 0.933 | T | G | 0.267 | 135 569 | 0.019 | 0.004 | 1.01E-07 | 0.020 | 0.004 | 5.01E-08 |  |
|  |  |  |  |  |  |  |  |  |  |  |  |  |  |
|  |  |  |  |  |  |  |  |  |  |  |  |  |  |
|  |  |  |  |  |  |  |  |  |  |  |  |  |  |
|  |  |  |  |  |  |  |  |  |  |  |  |  |  |
| **Key:** |  |  |  |  |  |  |  |  |  |  |  |  |  |
| SNP = SNP from main GWAS |  |  |  |  |  |  |  |  |  |  |  |  |  |
| Proxy SNP = Proxy SNP used in mtCOJO | |  |  |  |  |  |  |  |  |  |  |  |  |
| ld = LD between SNP and proxy, according to 1000G European populations | | | |  |  |  |  |  |  |  |  |  |  |
| A1 = allele 1 (coded allele) |  |  |  |  |  |  |  |  |  |  |  |  |  |
| A2 = allele2 |  |  |  |  |  |  |  |  |  |  |  |  |  |
| A1 freq = A1 allele frequency |  |  |  |  |  |  |  |  |  |  |  |  |  |
| Beta_cojo: Beta value conditioned on summary data from the PGC-PTSD major depressive disorder (MDD) GWAS, using multi-trait conditional and joint analysis (mtCOJO) | | | | | | | | | | | |  |  |
| SE_cojo: Standard error of beta conditioned on MDD | | |  |  |  |  |  |  |  |  |  |  |  |
| P_cojo: p-value of beta conditioned on MDD | |  |  |  |  |  |  |  |  |  |  |  |  |
|  |  |  |  |  |  |  |  |  |  |  |  |  |  |
| ¹As the summary statistics did not fully overlap between the MDD and childhood maltreatment GWAS. The most significant proxy SNP was used in mtCOJO. | | | | | | | | | | |  |  |  |
| ²To obtain beta values, applied transformation to Z scores, Z * SD of childhood maltreatment / sqrt (2 * N * MAF * (1 - MAF)), | | | | | | | |  |  |  |  |  |  |
| that assumed SD of childhood maltreatment was 0.83 (this is the SD of childhood maltreatment in the MRS sample) | | | | | | |  |  |  |  |  |  |  |
