# Supplementary material for: Genomic influences on self-reported childhood maltreatment
Source: Transl Psychiatry. 2020 Jan 27;10:38. doi: 10.1038/s41398-020-0706-0 (PMC7026037; doi:10.1038/s41398-020-0706-0)
Supplement: Supplementary file 5 — Supplementary Figures [file 41398_2020_706_MOESM5_ESM.docx]

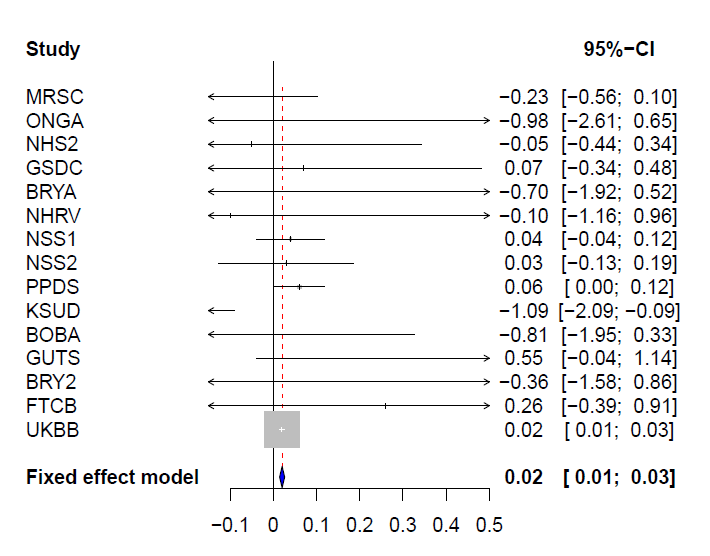


Supplementary Figure 1: Forest plot of the hit rs142346759 (chr3), showing effect sizes for each of the included GWAS in the meta-analysis.


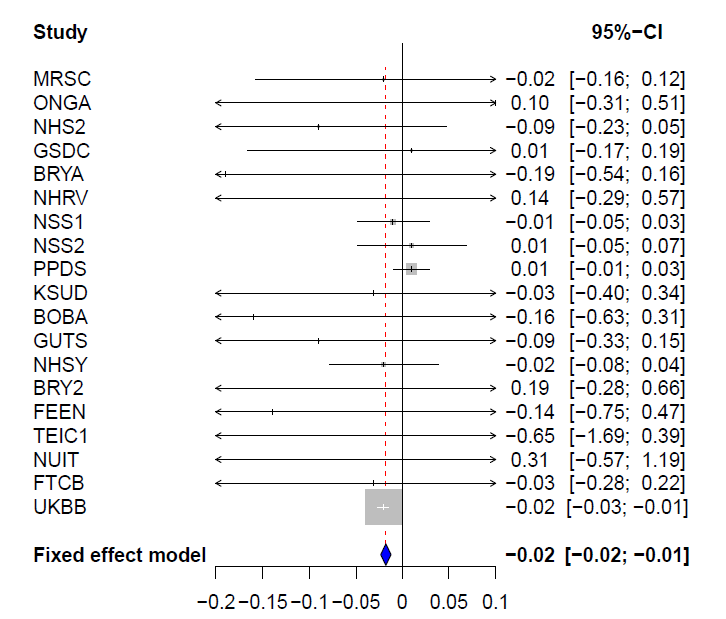


Supplementary Figure 2: Forest plot of the hit rs10262462 (chr7), showing effect sizes for each of the included GWAS in the meta-analysis.


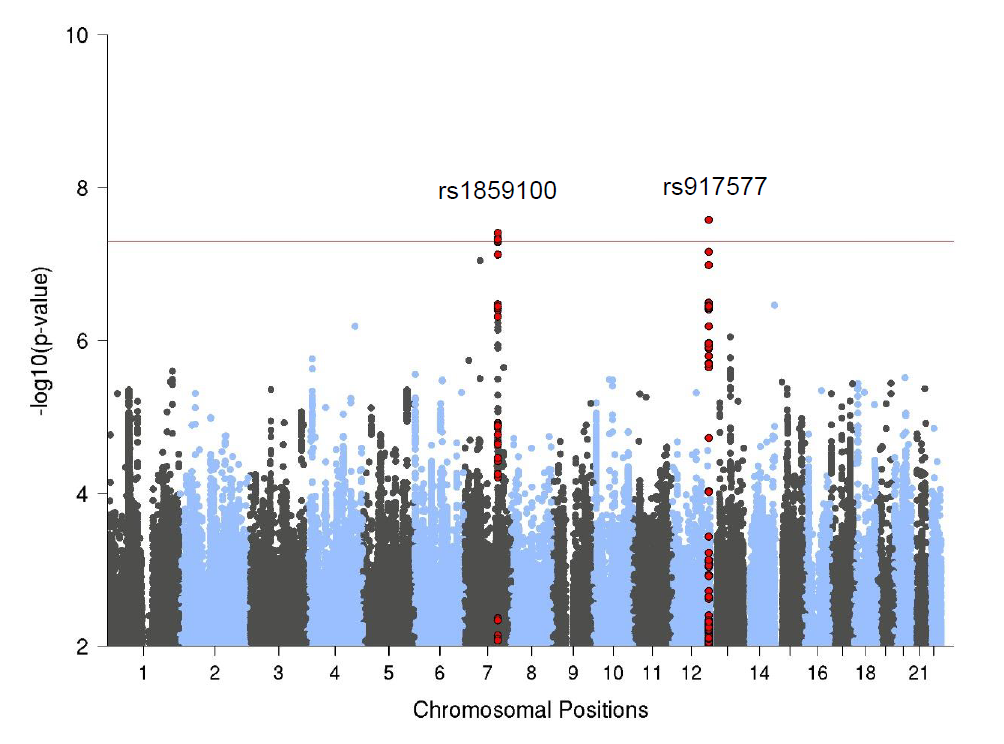


Supplementary Figure 3: Manhattan plot of the GWAS meta-analysis (UKBB and PGC1.5) for childhood maltreatment, showing the top variants. The horizontal line represents genome-wide significance at p<5x10^-8^.


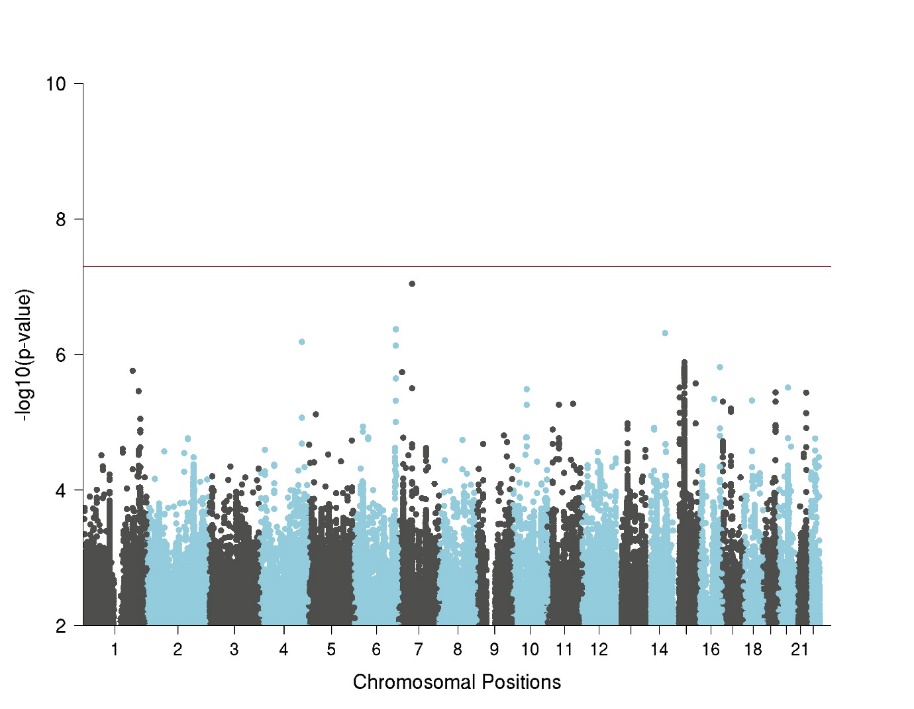


Supplementary Figure 4: Manhattan plot of PGC1.5 GWAS for childhood maltreatment. The horizontal line represents genome-wide significance at p<5x10^-8^.


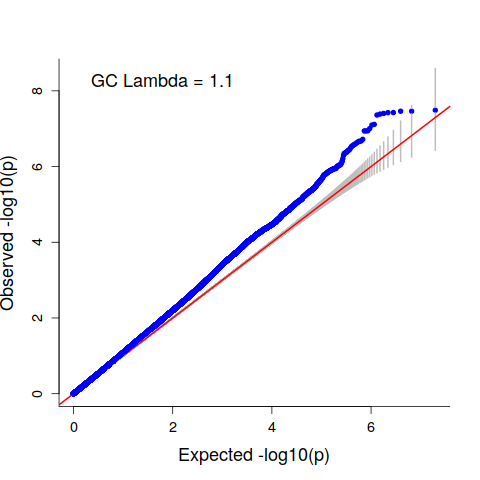


Supplementary Figure 5: Quantile-quantile (QQ) plots of expected versus observed -log_10_ p-values for genome-wide association studies (GWAS) for childhood maltreatment in the UKBB.


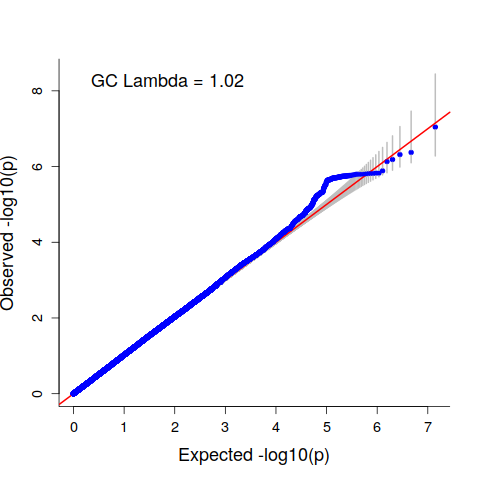


Supplementary Figure 6: Quantile-quantile (QQ) plots of expected versus observed -log_10_ p-values for PGC1.5 results.


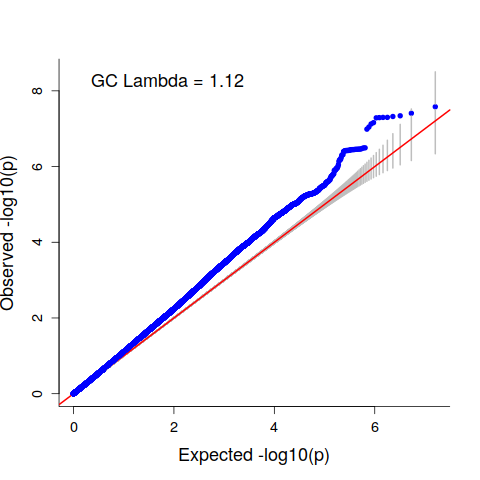


Supplementary Figure 7: Quantile-quantile (QQ) plots of expected versus observed -log_10_ p-values for the meta-analysis results.


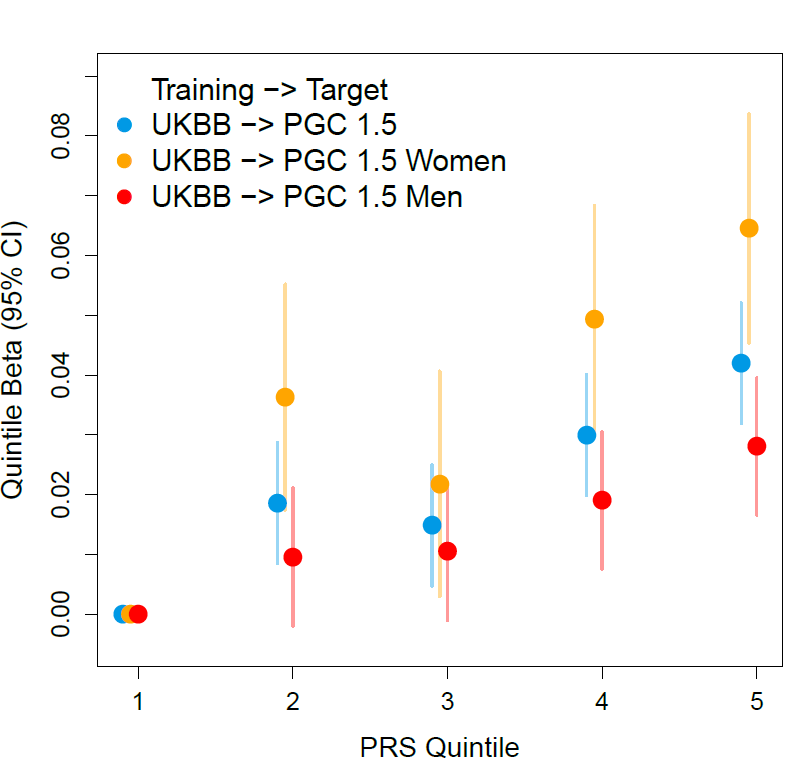


Supplementary Figure 8: Genetic risk score (PRS) predictions for childhood maltreatment. Using the UK Biobank as the discovery sample, effect sizes (Beta) for childhood maltreatment per PRS quantile relative to the first quantile show a significant increase in PGC1.5 childhood maltreatment target samples. For example, PGC1.5 subjects in the 5th quantile have childhood maltreatment scores 0.042 points higher (on a scale of childhood maltreatment ranging from 0 to 1) than PGC 1.5 subjects in the lowest quantile. PGC1.5 women reported significantly more childhood maltreatment than men. Error bars indicate 95% confidence limits.
